# Lesser prairie‐chicken dispersal after translocation: Implications for restoration and population connectivity

**DOI:** 10.1002/ece3.10871

**Published:** 2024-01-31

**Authors:** Liam A. Berigan, Carly S. H. Aulicky, Elisabeth C. Teige, Daniel S. Sullins, Kent A. Fricke, Jonathan H. Reitz, Liza G. Rossi, Kraig A. Schultz, Mindy B. Rice, Evan Tanner, Samuel D. Fuhlendorf, David A. Haukos

**Affiliations:** ^1^ Kansas Cooperative Fish and Wildlife Research Unit, Division of Biology Kansas State University Manhattan Kansas USA; ^2^ Kansas Department of Wildlife and Parks Emporia Kansas USA; ^3^ Colorado Parks and Wildlife Lamar Colorado USA; ^4^ Colorado Parks and Wildlife Steamboat Springs Colorado USA; ^5^ Kansas Department of Wildlife and Parks Elkhart Kansas USA; ^6^ U.S. Fish and Wildlife Service, National Wildlife Refuge System Fort Collins Colorado USA; ^7^ Department of Rangeland and Wildlife Sciences, Caesar Kleberg Wildlife Research Institute Texas A&M University Kingsville Texas USA; ^8^ Natural Resource Ecology & Management Oklahoma State University Stillwater Oklahoma USA; ^9^ U.S. Geological Survey, Kansas Cooperative Fish and Wildlife Research Unit Kansas State University Manhattan Kansas USA; ^10^ Present address: Native Prairies Association of Texas San Marcos Texas USA; ^11^ Present address: Department of Horticulture and Natural Resources Kansas State University Manhattan Kansas USA

**Keywords:** connectivity, Conservation Reserve Program, dispersal, prairie grouse, translocation, *Tympanuchus pallidicinctus*

## Abstract

Conservation translocations are frequently inhibited by extensive dispersal after release, which can expose animals to dispersal‐related mortality or Allee effects due to a lack of nearby conspecifics. However, translocation‐induced dispersals also provide opportunities to study how animals move across a novel landscape, and how their movements are influenced by landscape configuration and anthropogenic features. Translocation among populations is considered a potential conservation strategy for lesser prairie‐chickens (*Tympanuchus pallidicinctus*). We determined the influence of release area on dispersal frequency by translocated lesser prairie‐chickens and measured how lesser prairie‐chickens move through grassland landscapes through avoidance of anthropogenic features during their dispersal movements. We translocated 411 lesser prairie‐chickens from northwest Kansas to southeastern Colorado and southwestern Kansas in 2016–2019. We used satellite GPS transmitters to track 115 lesser prairie‐chickens throughout their post‐release dispersal movements. We found that almost all lesser prairie‐chickens that survived from their spring release date until June undergo post‐translocation dispersal, and there was little variation in dispersal frequency by release area (96% of all tracked birds, 100% in Baca County, Colorado, 94% in Morton County, Kansas, *n* = 55). Dispersal movements (male: 103 ± 73 km, female: 175 ± 108 km, *n* = 62) led to diffusion across landscapes, with 69% of birds settling >5 km from their release site. During dispersal movements, translocated lesser prairie‐chickens usually travel by a single 3.75 ± 4.95 km dispersal flight per day, selecting for steps that end far from roads and in Conservation Reserve Program (CRP) grasslands. Due to this “stepping stone” method of transit, landscape connectivity is optimized when <5 km separates grassland patches on the landscape. Future persistence of lesser prairie‐chicken populations can be aided through conservation of habitat and strategic placement of CRP to maximize habitat connectivity. Dispersal rates suggest that translocation is better suited to objectives for regional, rather than site‐specific, population augmentation for this species.

## Graphical abstract

Lesser prairie‐chickens translocated to southeastern Colorado and southwestern Kansas experienced almost universal dispersal away from the release site (96%). During dispersal movements, lesser prairie‐chickens selected for steps that ended far from roads and in Conservation Reserve Program grasslands. Dispersal rates suggest that translocation is better suited to regional, rather than site‐specific, population augmentation for this species.

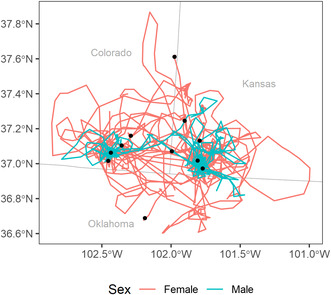

## 1 INTRODUCTION

Translocation is the act of anthropogenically moving individuals from one area to another and is typically used to introduce, reintroduce, or supplement populations in areas isolated from natural immigration (IUCN/SSC, 2013). While criteria for translocation success may vary, frequent criteria include the establishment of self‐sustaining populations and an increase in population size, demographic rates, or genetic diversity (Acevedo et al., 2023; Griffith et al., 1989; Nilsson et al., 2023). However, translocation success can be inhibited when animals act abnormally upon introduction to a novel environment, such as during dispersal away from the release site (Berger‐Tal et al., 2020). Post‐translocation dispersal can expose animals to dispersal‐related mortality (Yoder et al., 2004) or Allee effects due to a lack of nearby conspecifics (Armstrong & Wittmer, 2011), thereby negatively affecting the success of the translocation.

Though dispersal movements can complicate translocation efforts, they provide an opportunity for quantifying landscape connectivity by tracking large numbers of dispersing individuals moving across novel landscapes. Landscape connectivity, defined as the extent to which the landscape aids or deters movement among patches, is a critical aspect for preserving the viability of at‐risk species (Haddad et al., 2015; Taylor et al., 1993). At large spatial scales, landscape connectivity enables the exchange of individuals and genetic material among populations across a species' range, resulting in increased genetic diversity, range expansion, and recolonization of previously occupied habitats (Epps et al., 2005; Gil‐Tena et al., 2013; Smith et al., 2021). By facilitating immigration, landscape connectivity can aid regional recovery of populations after stochastic events and species range shifts in response to climate change (Rudnick et al., 2012; Sarremejane et al., 2021). At smaller spatial scales, landscape connectivity allows dispersers to reinforce, reestablish, or colonize nearby available habitat patches and assess relative habitat quality, facilitating the persistence of metapopulations dependent on immigrant input from source populations for population persistence (Pulliam, 1988).

The lesser prairie‐chicken (*Tympanuchus pallidicinctus*) is a grassland‐obligate species of conservation concern that is endemic to the southern Great Plains of the United States (Boal & Haukos, 2016). Lesser prairie‐chickens engage in a lek breeding system that is central to much of their life history; lesser prairie‐chickens stay near leks year‐round and typically nest within 3.2 km of a lek (Boal & Haukos, 2016). Landscape connectivity facilitates the flow of genetic material and population exchange within and across the four separate ecoregions that comprise the species' currently occupied range (Appendix S1: Figure S1; Westemeier et al., 1998, McDonald et al., 2014). Since the late 19th century, lesser prairie‐chickens have been declining in abundance and occupying range due to reductions in grassland habitat from agricultural conversion and woody plant encroachment (Fuhlendorf et al., 2002; Rodgers, 2016). Lesser prairie‐chickens require wide expanses of contiguous grassland for their persistence; reductions in grassland habitat can impede their ability to recover from drought events (Ross et al., 2016a). Reductions in grassland cover have been partially offset by the U.S. Department of Agriculture (USDA) Conservation Reserve Program (CRP), which began in 1985 and provides incentives for the conversion of cropland into grassland for 10–15 year contract periods (Spencer et al., 2017; Stubbs, 2014).

Translocation is a common technique in prairie grouse management; numerous prairie grouse translocations have occurred in the last few decades, often with anecdotal observations of extreme dispersal movements after translocation (Coates et al., 2006; Hamerstrom Jr & Hamerstrom, 1951; Vogel, 2015). However, widespread monitoring of translocations is rare, as are evaluations of how these dispersal movements affect the efficacy of translocations (Snyder et al., 1999; Teige et al., 2023). Dispersal following translocation can negatively affect translocation success due to mortality and diffusion throughout the release site, especially for sage and other prairie grouse (i.e., *Centrocercus* spp, *Tympanuchus* spp; Reese & Connelly, 1997, Snyder et al., 1999). To ameliorate these effects, past translocations have attempted to reduce the proportion of dispersing prairie grouse through release at leks or near nesting habitat (Coates et al., 2006). Despite consideration of translocation as a tool for lesser prairie‐chicken restoration efforts (Berigan et al., 2022; Solomon, 2022), there has been little study of the rate or extent of dispersal of lesser prairie‐chickens after translocation, and whether lesser prairie‐chickens follow dispersal patterns observed in other species of prairie grouse. Anecdotal observations report the disappearance of lesser prairie‐chickens from the release site after translocation and cite dispersal as a likely cause for translocation failure (a comprehensive overview of past lesser prairie‐chicken translocation efforts is in Appendix S2).

In native populations, lesser prairie‐chicken dispersal movements are generally defined as one‐way movements that result in >5 km displacement from a bird's home range, as these movements are longer than would be expected during typical daily activity (Earl et al., 2016; Haukos & Zavaleta, 2016). Dispersal movements typically occur during late spring and summer (net displacement: $\bar{x}$ = 16.18 km, SE = 2.77 km), with female lesser prairie‐chickens being responsible for the most frequent movements (males dispersing per year: 2.1%–5.9%, females: 13.1%–48.4%; Earl et al., 2016). Anthropogenic structures can repel lesser prairie‐chickens during dispersal movements at distances up to 350 m for oil and gas wells and 5.4 km for electrical transmission lines (Peterson et al., 2020). Anthropogenic barriers can restrict movements by lesser prairie‐chickens in a similar manner to habitat fragmentation, resulting in reduced landscape connectivity. However, it is still unclear how land cover types and configurations affect lesser prairie‐chicken dispersal movements in novel landscapes, and how landscape composition works in concert with anthropogenic barriers to limit lesser prairie‐chicken population connectivity.

We studied movements and land cover selection by dispersing lesser prairie‐chickens following translocation of birds from the Short‐Grass Prairie/CRP Mosaic Ecoregion to the Sand Sagebrush (*Artemisia filifolia*) Prairie Ecoregion in 2016–2019. We examined whether lesser prairie‐chickens translocated to two ecologically distinct areas would undergo similar dispersal movements after translocation; as a subobjective, we measured whether female dispersal movements might be constrained by their usual tendency to nest within 3.2 km of a lek (Boal & Haukos, 2016). When dispersal occurred following translocation, we evaluated how lesser prairie‐chickens moved across novel landscapes in relation to land cover classes and potential anthropogenic obstacles to assess how these features affect landscape connectivity. Based on prior research suggesting that dispersal frequency varies among translocated individuals based on habitat near the release site (Coates et al., 2006), we hypothesized that post‐translocation dispersal would not be a universal trait for lesser prairie‐chickens, as post‐translocation dispersal frequency would instead vary between ecologically distinct release sites. We predicted that lesser prairie‐chickens released in Baca County, Colorado, which is composed of CRP and short‐grass prairie and more closely resembled landscapes where source birds were captured, would disperse at a lower rate than those released in Morton County, Kansas, which included a novel habitat type, sand sagebrush prairie. Based on studies that demonstrate that lesser prairie‐chickens are dependent on grassland habitat throughout much of their life cycle and avoid anthropogenic obstacles (Boal & Haukos, 2016), we also hypothesized that lesser prairie‐chickens would move through grassland and avoid anthropogenic features during their dispersal movements. We predicted that a step selection function (Fortin et al., 2005) would find selection for steps that end in CRP grassland and selection against steps that cross or end near anthropogenic features during lesser prairie‐chickens' post‐translocation dispersal movements.

## 2 METHODS

### 2.1 Study area

We translocated lesser prairie‐chickens from the Short‐Grass Prairie/CRP Mosaic Ecoregion in northwestern Kansas to the Sand Sagebrush Prairie Ecoregion in southwestern Kansas and southeastern Colorado (Appendix S1: Figure S1). The two counties where we released lesser prairie‐chickens (Baca County, Colorado [662,260 ha], and Morton County, Kansas [189,069 ha]) were composed of row‐crop agriculture (31.8% Baca, 47.7% Morton), CRP grasslands (16.6% Baca, 17.7% Morton), and native prairies (49.8% Baca, 33.9% Morton). Oil and gas exploration occurs in both counties, with 113 active wells on record in Baca County (Colorado Oil and Gas Conservation Commission, 2020) and 2139 active wells on record in Morton County (Kansas Geological Survey, 2020). The vegetation communities found in Baca and Morton counties included both short‐grass and sand sagebrush (*Artemisia filifolia*) prairies. Short‐grass prairie was dominated by blue grama (*Bouteloua gracilis*) and buffalograss (*B. dactyloides*), while sand sagebrush prairie was primarily composed of sand sagebrush, sand dropseed (*Sporobolus cryptandrus*), western ragweed (*Ambrosia psilostachya*), and blue grama (Berigan et al., 2022). Mean monthly temperatures during the study period (2016–2019) ranged from 0.3 to 27.3°C in Elkhart, Kansas, and from −1.4 to 25.6°C in Springfield, Colorado (High Plains Regional Climate Center, 2023). Annual precipitation during the study period ranged from 40.9 to 67.0 cm in Elkhart, Kansas, and 31.0–52.2 cm in Campo, Colorado. The historical average annual precipitation is 46 cm in Elkhart, Kansas (SD: 12 cm, range: 23–76 cm, date range: 1900–2021) and 38 cm in Springfield, Colorado (SD: 11 cm, range: 19–68 cm, date range: 1916–1970; High Plains Regional Climate Center, 2023).

The USDA‐Forest Service manages 65,437 ha of land as part of the Comanche and Cimarron National Grasslands in Baca (12.2%) and Morton (23.0%) counties, respectively, with a focus on providing multi‐use opportunities for grazing, energy exploitation, and wildlife recreation. Translocated lesser prairie‐chickens were released on Comanche and Cimarron National Grasslands. Although both counties are within the Sand Sagebrush Prairie Ecoregion, different soil types, precipitation, and management have led to distinct differences in vegetation composition between the counties and National Grasslands (Berigan et al., 2022). The Cimarron National Grassland, in particular, has a greater proportion of sand sagebrush within its cover types ($\bar{x}$ = 12%, range: 0%–37%) than the Comanche National Grassland ($\bar{x}$ = 5%, range: 3%–6%); grasslands on the Cimarron frequently have a greater forb composition than their Comanche counterparts (Cimarron: $\bar{x}$ = 32%, range: 11%–56%; Comanche: $\bar{x}$ = 26%, range: 23%–30%; Berigan et al., 2022). Public and private rangelands in Morton County often have a greater proportion of sod‐grasses, with low value for lesser prairie‐chicken nesting habitat, than Baca County (Morton: $\bar{x}$ = 32%, range: 2%–71%; Baca: $\bar{x}$ = 28%, range: 18%–42%; Berigan et al., 2022). Although tall and mid‐grasses with high nesting value are more common in Morton County CRP (24%) than Baca County CRP (11%; Berigan et al., 2022), vegetation composition of CRP is similar between the counties. CRP patch sizes are on average smaller in Morton County ($\bar{x}$ = 64.7 ha, median = 48.5 ha, SD = 84.8 ha) than in Baca County ($\bar{x}$ = 121.4 ha, median = 65.0 ha, SD = 160.8 ha).

### 2.2 Translocation and monitoring

Between fall 2016 and spring 2019, we translocated 411 lesser prairie‐chickens captured on leks to the Cimarron and Comanche National Grasslands using a hard release technique (not including measures that would facilitate a gradual transition to the release site; De Milliano et al., 2016). The initial fall 2016 release was primarily males (26 males, 1 female); all subsequent releases were in spring and had similar numbers of males and females (Berigan et al., 2022; Teige et al., 2023). We equipped birds translocated during fall 2016 and spring 2017–2019 with 11‐g bib‐style very‐high‐frequency (VHF) transmitters. We used RI‐2B VHF transmitters from Holohil Systems Ltd. (Carp, ON, Canada) in 2016 and 2017, and used Series A3900 VHF transmitters from Advanced Telemetry Systems (Isanti, MN, USA) in 2018 and 2019. Additionally, during 2018 and 2019, we deployed rump‐mounted 22‐g Satellite Platform Transmitting Terminal (SAT‐PTT) GPS transmitters (PTT‐100; Microwave Telemetry, Columbia, MD, USA) on translocated birds (a total of 115 birds with GPS transmitters and 279 birds with VHF transmitters). The final 17 translocated birds were not given a GPS or VHF tag due to a lack of supply; these birds were therefore excluded from monitoring.

**FIGURE 1:**
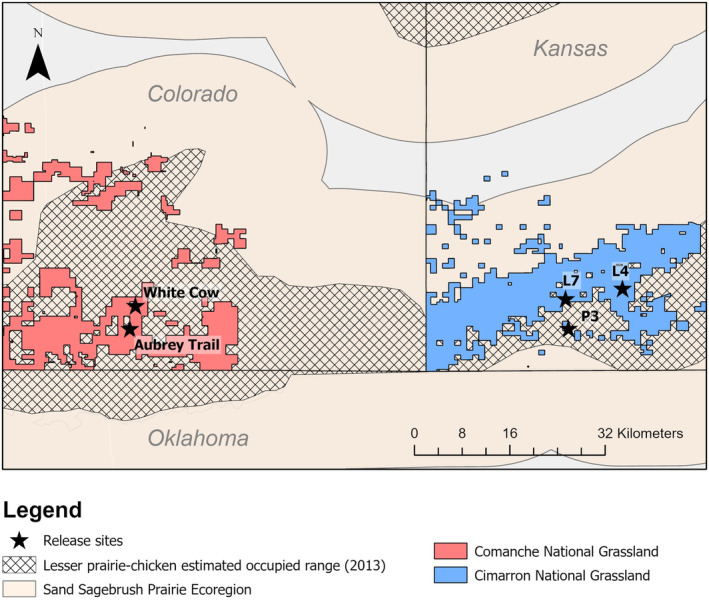
Release sites for translocated lesser prairie‐chickens (*Tympanuchus pallidicinctus*) on the U.S. Department of Agriculture‐Forest Service Cimarron and Comanche National Grasslands in Kansas and Colorado, respectively. Translocated birds were released at Aubrey Trail and P3 in 2016–2017, White Cow, P3, and L7 in 2018, and White Cow and L4 in 2019.

We initially released birds on either the Cimarron or Comanche National Grasslands in 2016 and 2017 in areas chosen for their proximity to presumed high‐quality nesting habitat, determined based on plant species composition, visual obstruction, and historical leks. We adjusted release sites in 2018 and 2019 once translocated birds began lekking to ensure that birds were released near active lekking or previous nesting sites. The release site on the Comanche National Grasslands in 2016–2017 was at the Aubrey Trail lek, which had active lekking through spring 2016. In spring 2018 and 2019, we moved the release site on the Comanche National Grasslands to the historic White Cow lek, which was re‐established as a lekking site in 2019. The release site on the Cimarron National Grassland in 2016–2017 was on USDA‐Forest Service land adjacent to the P3 lek, a small active lek to the south of the Cimarron River. In 2018, we released birds translocated to the Cimarron National Grasslands jointly at P3 (which became inactive in 2018) and a new release site at the inactive historical L7 lek, chosen because of its proximity to presumed quality nesting habitat on the Cimarron National Grassland. In 2019, we released all birds translocated to the Cimarron National Grassland at the L4 site (Figure 1; Teige et al., 2023).

We attempted to monitor birds equipped with VHF transmitters at least three times per week, but due to extensive dispersal movements following release, many VHF birds went missing for a period of one or more months, with some birds never located (Teige et al., 2023). A Colorado Parks and Wildlife plane was used monthly (May–July) to relocate missing VHF birds via aerial telemetry. We monitored birds equipped with satellite transmitters remotely, with a GPS location recorded at ±18 m accuracy every 2 h between 1100 and 0500 UTC (0600 and 0000 Central Daylight Time) and uploaded to an ARGOS satellite every 3 days. When tagged birds congregated in an area, we surveyed that area at sunrise during spring to determine if lekking was occurring. We surveyed all active and historic leks on the Cimarron and Comanche National Grasslands, as well as leks established or visited by translocated birds off the National Grasslands, at least twice each spring in 2016–2019 for attendance by translocated and native lekking males. We used known lek locations to quantify the number of leks that satellite‐tagged birds visited (defined as having a satellite location within 500 m of a lek) during their dispersal movement and identify whether nesting patterns near leks deviated from what was expected for native populations.

### 2.3 Dispersal frequency and characteristics

Long‐distance movements are defined in native lesser prairie‐chicken populations as movements that cause the bird to leave an area within a 5‐km radius of its home range, either temporarily or permanently, as movements >5 km are longer than would be expected during typical daily activity (Earl et al., 2016; Haukos & Zavaleta, 2016). Earl et al. (2016) further refine long‐distance movements into three categories based on whether they were unidirectional (dispersal), connected multiple distinct home ranges that the bird used during different portions of the year (round‐trip movements), or resulted in the bird returning to the same home range it departed from without establishing any new home range (foray loop). As a visual inspection of our data found no evidence of clear delineations of movements that would support the inclusion of round‐trip movements or foray loops as categories, we categorized all long‐distance movements occurring post‐translocation as dispersal movements. We herein define post‐translocation dispersal as any movement that results in a lesser prairie‐chicken either temporarily or permanently leaving an area within a 5‐km radius of its release site.

Because we were unable to locate birds with VHF transmitters regularly during dispersal movements, only satellite birds could be used to delineate dispersal movements. We further subsetted our dataset to only include those birds that underwent movements that met the definition for a dispersal movement in the spring and summer following their release. We identified the beginning and end of these dispersal movements using satellite locations, subsampled to one location per day, to conduct a behavioral change point analysis using the fitHMM function in the moveHMM package and the partmod.ltraj function in the adehabitatLT package in R (Calenge, 2006; Michelot et al., 2016; R Core Team, 2023). The behavioral change point analysis aimed to determine the point at which a dispersing lesser prairie‐chicken transitioned from a dispersing state to a settled state (defined as no longer undergoing dispersal movements for the duration of the season) based on the lesser prairie‐chicken's daily step lengths and turn angles. We limited the behavioral change point analysis to satellite‐tagged birds that survived to reach a settled state, and further excluded those that did not have a clear transition between a dispersing and settled state or failed to converge on a single behavioral change point. We used the remaining behavioral change points to determine the total distance traveled, the net distance from the release site to the settlement point, and the time elapsed during the dispersal movement for each bird.

### 2.4 Distribution of nests in relation to leks

To determine the effect of dispersal on nest locations, we monitored the nesting effort of translocated female lesser prairie‐chickens from 2017 to 2019. We used weekly GPS updates to determine the nest initiation and termination dates, location, and fate of satellite‐tagged birds. We never intentionally flushed satellite‐tagged birds and checked nests only after location, and sensor data indicated that the female had either permanently left the nest or died on the nest (Lautenbach et al., 2019). We determined nest locations for VHF‐tagged birds by using radio telemetry to approach and visually confirm nest locations, monitoring nests daily using radiotelemetry from a point ~100 m away. We checked the VHF nest fate after the bird was detected off‐nest for 3 days in a row or once a mortality signal was detected. We evaluated nest locations to determine whether dispersal after translocation caused lesser prairie‐chickens to nest further from leks than expected for native populations (i.e., within 3.2 km; Boal & Haukos, 2016).

### 2.5 Step selection during dispersal

We used a step selection function to measure the effects of land cover and anthropogenic features on lesser prairie‐chicken movement during dispersal (Fortin et al., 2005). As each bird usually only had one dispersal movement per day, we resampled each movement trajectory used in the step selection analysis to a single location per day at 1700 Central Daylight Time (CDT), reflecting a bird's destination at the end of its daily flight. Each step in the analysis was therefore defined as a linear feature between points on two successive days. We organized the dependent variables for the step selection function into four suites: land cover at endpoint, distance from endpoint to obstacle, land cover along the step, and obstacle crossing. The land cover at endpoint category represented the land cover type present at the endpoint of a bird's step. We delineated land cover types using the 2018 and 2019 Cropland Data Layer (Boryan et al., 2011) and further delineated CRP using shapefiles obtained from the USDA Farm Service Agency reflecting CRP enrollment in 2014 (Kansas and Colorado) and 2016 (Oklahoma). The Cropland Data Layer includes four different developed categories, differentiated based on their impervious cover: open, low intensity, medium intensity, and high intensity. Due to a low representation of steps ending in the latter three cover types in the dataset, we lumped the low, medium, and high intensity categories into a single category for further analysis. We ground‐truthed the locations of grassland patches using vegetation surveys of 299 randomly selected grassland patches in Baca and Morton counties (Berigan et al., 2022). Each land cover type was used as a binary predictor in the step selection function, with cropland (the most predominant cover type) used as the reference state. Five land cover types (forest, barren, water, wetland, and developed [low, medium, and high intensities]) were used fewer than five times each among all birds and thus excluded from analysis. The non‐reference cover types that were retained for analysis included CRP, non‐CRP grassland, shrubland, and developed (open). The non‐CRP grassland and shrubland categories included publicly and privately owned working grasslands (i.e., regularly grazed rangelands), which were primarily composed of native plant species (Berigan et al., 2022). We *z*‐transformed all variables before analysis to ensure that beta coefficients were comparable between suites.

Variables in the distance from endpoint to obstacle category measured the distance from the endpoint of a step to roads, electrical transmission lines, and oil wells. We obtained locations of primary roads (hereafter streets), secondary roads (hereafter highways), and transmission lines from the U.S. Census Bureau's 2010 TIGER dataset (U.S. Census Bureau, 2015). We obtained locations of oil wells from the Kansas Geological Survey (2020), the Colorado Oil and Gas Conservation Commission (2020), and the Oklahoma Corporation Commission (2020). We filtered these locations to those oil and gas wells that were not yet plugged, as plugging involves the removal of well infrastructure and remediation of the site. We used a natural logarithm to log‐transform all variables in the distance from endpoint to obstacle category to normalize their distributions before they were *z*‐transformed.

Variables in the land cover along step category measured proportional land cover along each step, using the same set of land cover types as for the land cover at endpoint analysis. We assumed that each 24‐h step was a straight line between the start and endpoints of each step and extracted proportional land cover along each linear step using the extract_covariates_along function in the amt package (Signer et al., 2019). Variables in the obstacle crossing category measured when a bird crossed a linear feature such as a street, highway, or transmission line during a given step. Due to the rarity of crossing large obstacles, such as highways and transmission lines, we recorded the presence or absence of all obstacle crossings as a binary variable, with any step with at least one obstacle crossing recorded as 1, and any step with no obstacle crossings recorded as 0.

We fit a step selection function using the dispersal movements (defined as steps between the date of release and the date of settlement in the behavioral change point analysis) of satellite‐tagged lesser prairie‐chickens for which we had at least 2 weeks of data and could effectively delineate dispersal movements. We elected to combine both sexes for the step selection function to increase the statistical power of our analysis; sex‐specific results are available in Appendix S3. Step selection functions use the distributions of observed step lengths and turn angles, simplified into gamma and von Mises distributions, respectively, to generate hypothetical “available” steps in each timestep to compare to the observed “used” step (Fortin et al., 2005). We chose to generate nine random available steps for each used step using the random_steps function in the amt package (Signer et al., 2019). The number of steps was chosen to provide a meaningful representation of available trajectories on the landscape, while also using few enough steps to maintain computational feasibility (Thurfjell et al., 2014). We extracted variables from each of the four suites (land cover at endpoint, distance from endpoint to obstacle, land cover along the step, and obstacle crossing) to each used and available step. We then compared the predictive capacities of each of these *z*‐transformed variables by testing a set of generalized linear mixed models using the glmmTMB package (Brooks et al., 2017). We used this package to incorporate random slopes for each individual bird following Muff et al. (2020), allowing population‐level inference while still accounting for differences in selection among individuals. Each model included a variable, an individual‐specific random slope for the variable, a step‐specific random intercept, and a natural logarithm of the step length. We incorporated the natural logarithm of the step length to compensate for strong selection for shorter step lengths, which were more frequent than the occasional large step that could be simulated by the step selection function. The null model for this analysis included both the step‐specific random intercept and the natural logarithm of the step length to give it comparable predictive capacity to the test models. We tested each of these models with a fixed variance of 1000 for the step‐specific random intercept following Muff et al. (2020). To determine which variables were most influential in step selection, we ranked models within each suite using Akaike Information Criteria scores, corrected for small sample sizes (AIC_c_; Burnham & Anderson, 1998). The top model from each of these suites was included in an ensemble suite, which compared the predictive capacity of the most informative models.

## 3 RESULTS

### 3.1 Dispersal frequency and characteristics

During 2016–2019, 411 birds were translocated to the Cimarron and Comanche National Grasslands, 394 of them with transmitters. Of these, 115 birds were equipped with satellite transmitters, which allowed us to examine the full dispersal movement after release. Almost all satellite‐tagged birds that survived from their spring release dates (March 21st–April 19th) until June (*n* = 55) initiated a >5 km dispersal movement after release (100% in 2018, 91% in 2019; 96% overall). The rate of dispersal from the Comanche National Grassland (100%) was similar to the rate of dispersal from the Cimarron National Grassland (94%).

Further measurements of lesser prairie‐chicken dispersal movements (except for habitat measurements; see section 3.3) only used a subset of data from birds that were known to have completed their dispersal movements. Of 115 satellite‐tagged lesser prairie‐chickens, we excluded 40 from consideration due to mortality or transmitter failure prior to the end of their dispersal movements. We excluded a further 12 birds that did not have a clear transition between a dispersing and settled state or failed to converge on a single behavioral change point, leaving 62 birds available to analyze the time of dispersal movements, dispersal distance, duration, movement patterns, and displacement from the release site.

**FIGURE 2:**
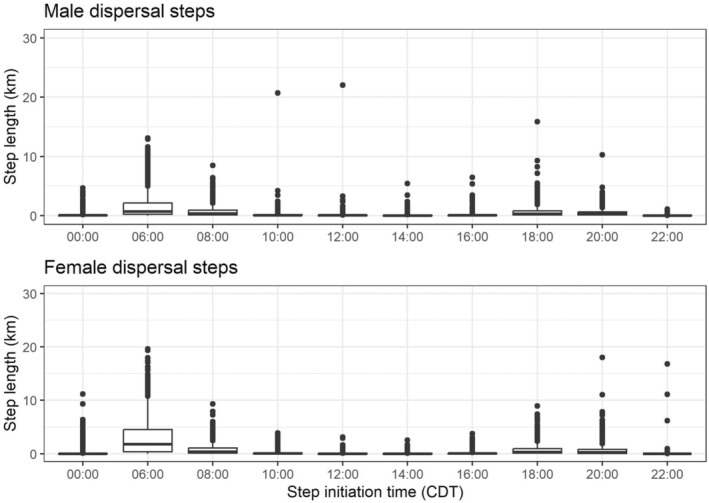
Step lengths (km) by timestep for 62 lesser prairie‐chickens (*Tympanuchus pallidicinctus*) equipped with SAT‐PTT transmitters during their dispersal after release in southeastern Colorado and southwestern Kansas in 2018–2019. Transmitters were programmed to take one location every 2 h from 0600 to 0000 Central Daylight Time. The timestep beginning at 0600 reflects movements that take place during morning lekking, with a less intense lekking period taking place again at sunset during the timestep beginning at 1800. Five outliers from female birds are not displayed here: a 38‐km movement originating at 1400 CDT, a 45‐km movement initiating at 0800, a 57‐km movement originating at 1200, a 65‐km movement originating at 0000, and a 67‐km movement originating at 0800. Boxplots indicate the median and interquartile range, while whiskers extend to the largest/smallest value within 1.5 times the interquartile range.

Although lesser prairie‐chickens were active and moving during most daylight hours, larger dispersal movements were usually limited to two time periods. Both males and females were most likely to undergo large dispersal movements from 0600 to 0800 CDT, which is the timestep that includes sunrise and the majority of lekking activity (Figure 2). The next most frequent time for dispersal movements was from 1800 to 2000, when sunset occurs and there is a small increase in lekking activity. During the midday hours (1000–1600) and the evening hours (2200–0600), most birds were either stationary or undergoing short, non‐directional movements, likely indicating roosting and foraging, respectively.

**FIGURE 3:**
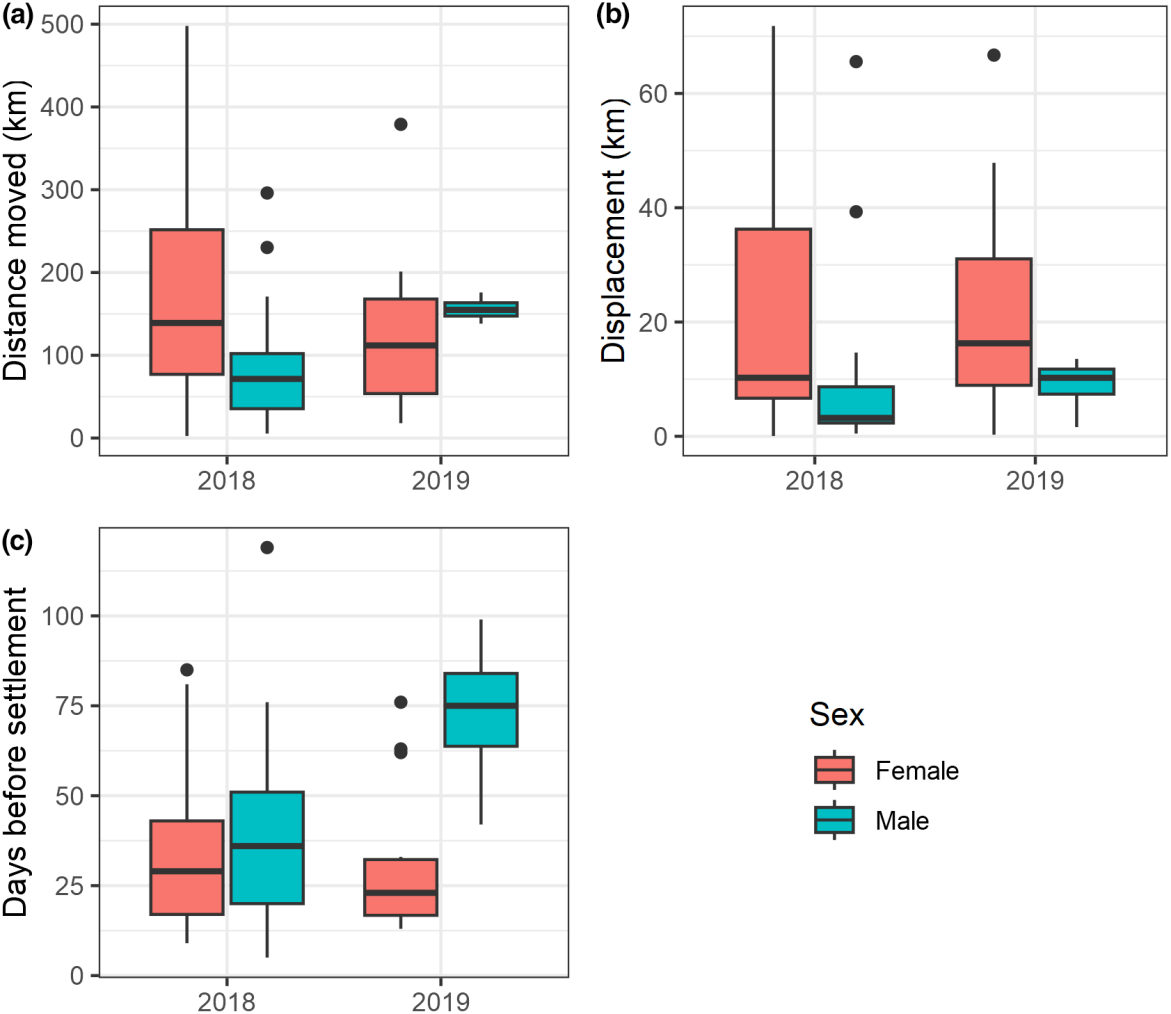
(a) Total distance moved between release and settlement for 62 satellite‐equipped lesser prairie‐chickens (*Tympanuchus pallidicinctus*) translocated to southwestern Kansas and southeastern Colorado in 2018 (*n* = 42) and 2019 (*n* = 20), representing the distance traveled during the dispersal period. (b) Distance from the release site to the settlement site (km), representing displacement from the release site at the conclusion of the dispersal movement. (c) The number of days between the release and settlement dates, representing the amount of time each translocated lesser prairie‐chicken spent dispersing. Boxplots indicate the median and interquartile range, while whiskers extend to the largest/smallest value within 1.5 times the interquartile range.

Lesser prairie‐chickens released in novel landscapes traveled hundreds of kilometers during their dispersals following release (length of track: female $\bar{x}$ = 175 km, SD = 108 km, range = 15–474 km, *n* = 41; male $\bar{x}$ = 103 km, SD = 73 km, range = 26–279 km, *n* = 21; Figure 3a), frequently displaying circular and occasionally recursive movements. Visual inspection of satellite tracks showed that lesser prairie‐chickens moved independently during dispersal, exhibiting no evidence of flocking despite being released at common locations. Following dispersal, 69% of all released birds settled >5 km from their release site. Sites where birds ceased their dispersal movements were frequently distant from the release site (net displacement: female $\bar{x}$ = 23 km, SD = 20 km, range = 0.7–69 km, *n* = 41; male $\bar{x}$ = 13 km, SD = 21 km, range = 0.5–64 km, *n* = 21; Figure 3b), but this distance was usually considerably less than the total movement distance. Dispersal movements started a few days after release ($\bar{x}$ = 2.3 days, range = 0–7 days, *n* = 62, excluding two dispersals post‐nesting), and were 1–2 months long (female $\bar{x}$ = 52 days, SD = 24 days, range = 15–100 days, *n* = 41; male $\bar{x}$ = 46 days, SD = 17 days, range = 15–75 days, *n* = 21; Figure 3c). Average daily step lengths were 2.50 ± 3.36 km for males, 4.77 ± 5.75 km for females, and 3.75 ± 4.95 km for both sexes.

**FIGURE 4:**
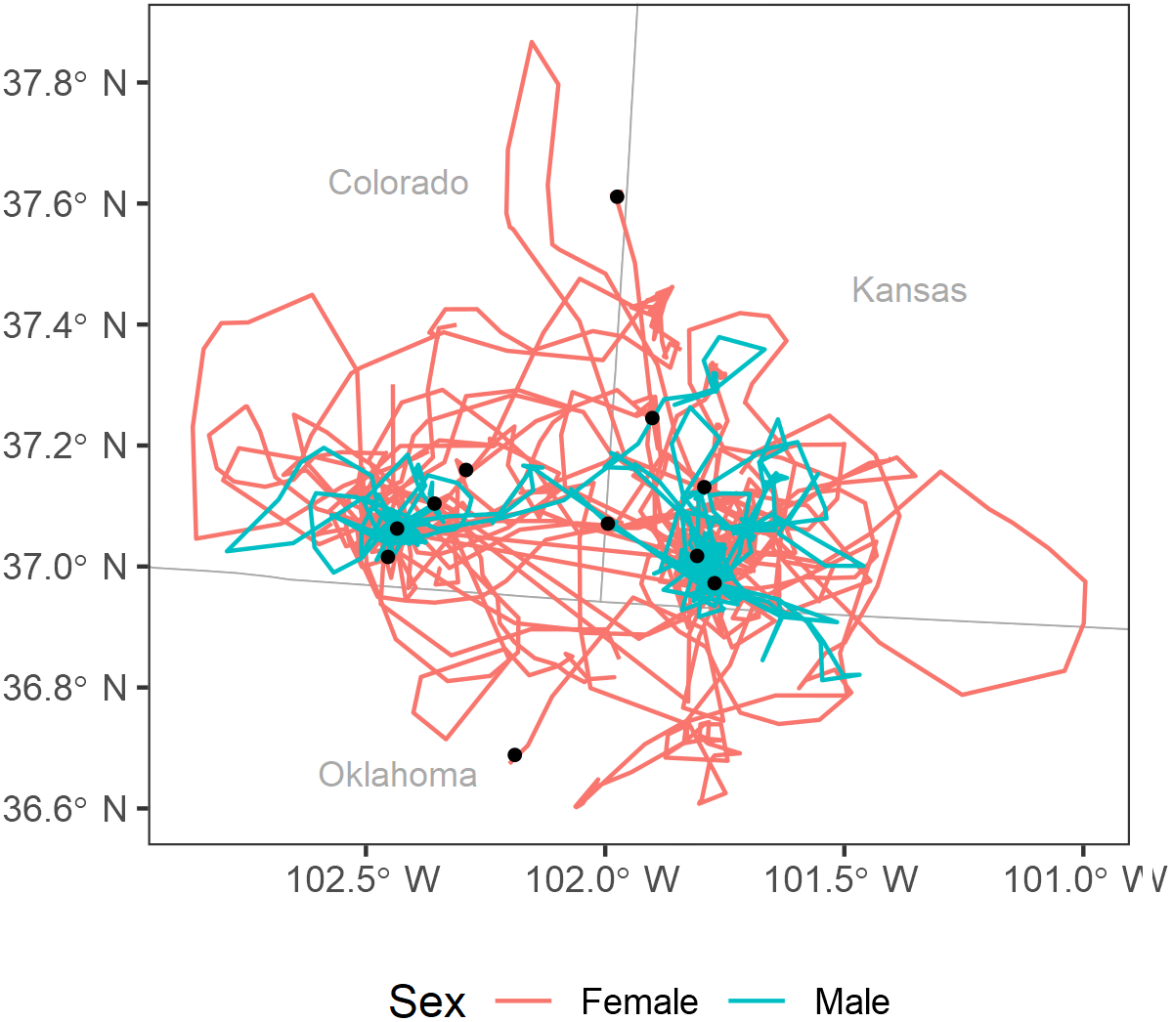
Dispersal trajectories of male (*n* = 20) and female (*n* = 42) lesser prairie‐chickens (*Tympanuchus pallidicinctus*) translocated to southeastern Colorado and southwestern Kansas in 2016–2019. Lesser prairie‐chicken females move further from leks (black dots) than males during dispersal.

Although males and females moved comparable distances during their dispersal (Figure 3a), movement patterns differed between sexes (Figure 4). Male lesser prairie‐chickens usually visited (defined as having a satellite location within 500 m of a lek) one or two known leks during their dispersal movement (males: $\bar{x}$ = 1.24 leks, SD = 0.89, range = 0–3, *n* = 21; females: $\bar{x}$ = 0.66 leks, SD = 0.73, range = 0–2, *n* = 41), and then settled near one of these leks at the conclusion of their dispersal. Females tended to encounter a smaller number of leks during dispersal than males and settled further from the nearest lek than males (male $\bar{x}$ = 2.91 km from the nearest lek, SD = 4.28 km, range = 0.03–14.27 km, *n* = 21; female $\bar{x}$ = 8.85 km from the nearest lek, SD = 8.82 km, range = 0–35.11 km, *n* = 41).

### 3.2 Distribution of nests in relation to leks

Female dispersal to areas distant from leks resulted in an atypical distribution of nests across the landscape. While almost all females in a native population would be expected to nest within 3.2 km of a lek (Boal & Haukos, 2016), translocated females frequently nested in areas with no leks nearby (distance to nearest lek: $\bar{x}$ = 7.9 km, SD = 8.9 km, *n* = 124; Appendix S4: Figure S3).

### 3.3 Step selection during dispersal

**FIGURE 5:**
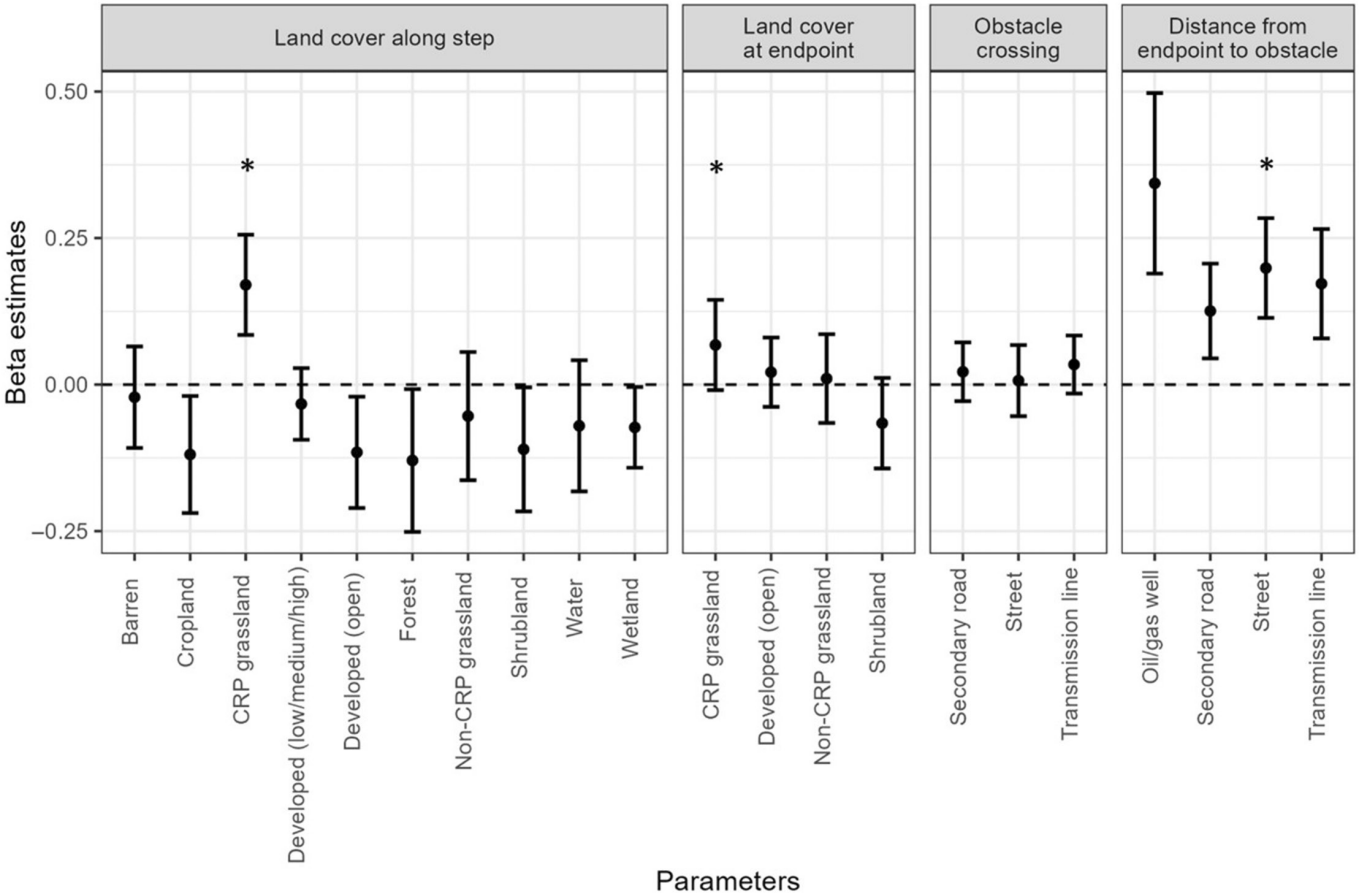
Beta estimates with 95% confidence intervals for all variables used as covariates in a step selection function (Fortin et al., 2005) used to determine how lesser prairie‐chickens (*Tympanuchus pallidicinctus*) select for landscape features during dispersal after release in southeastern Colorado and southwestern Kansas in 2016–2019. Beta estimates are extracted from single‐variable models with *z*‐scaled covariates. Positive estimates demonstrate selection for a variable, while negative estimates demonstrate avoidance. Note that selection for covariates in the “distance from endpoint to obstacle” category constitutes avoidance of the appropriate obstacle; for example, lesser prairie‐chickens select for steps whose endpoints are far from oil wells. Asterisks mark the most informative model in each suite, as determined using the Akaike Information Criterion (corrected for small sample sizes). The absence of an asterisk in a suite indicates that the null model was the most informative model.

The step selection analysis used data from a subset of individuals, for which we had at least 2 weeks of data and could effectively delineate dispersal movements. Of 115 satellite‐tagged birds, we excluded 22 that had <2 weeks of locations prior to mortality and removed a further 18 that did not have a clear transition between a dispersing and settled state or failed to converge on a single behavioral change point, leaving a sample size of 75 birds and 2371 daily movement steps for the step selection analysis. Model rankings demonstrated that most models were better supported than the null model in predicting dispersal steps (Table 1A–E). The only models that ranked below the null model were the three obstacle crossing models and the proportion of developed (low, medium, and high) landcover along a step. The top‐ranked model in the ensemble suite was CRP at the endpoint of the step (*β* = .068, SE = 0.039), which held 100% of the model weight. Lesser prairie‐chickens were 1.18× more likely to select steps that ended in CRP grassland. Several other covariates had larger beta estimates but were less informative than the top model (Figure 5). The proportion of the step that was composed of CRP grassland had a correlation of 0.63 with CRP at the endpoint of the step but produced a less informative model (ΔAIC_c_ = 15.08).

**TABLE 1:** (A–E) Model selection tables used to determine the effect of land cover and obstacles on lesser prairie‐chicken (*Tympanuchus pallidicinctus*) step selection during dispersal after translocation to Morton County, Kansas and Baca County, Colorado in 2018–2019.

Suite^a^	Model^b^	*K* ^c^	ΔAICc^d^	*w*_i_ ^e^
**A**				
Land cover along step	CRP	3	0	0.8
	Non‐CRP grassland	3	2.71	0.2
	Shrubland	3	22.57	0
	Developed (open)	3	44.12	0
	Log step length (null model)	2	49.61	0
**B**				
Land cover at endpoint	CRP	3	0	1
	Non‐CRP grassland	3	32.84	0
	Shrubland	3	32.85	0
	Developed (open)	3	59.23	0
	Log step length (null model)	2	64.69	0
**C**				
Obstacle crossing	Log step length (null model)	2	0	0.6
	Transmission line	3	2.19	0.2
	Highway	3	3.28	0.11
	Street	3	3.80	0.09
**D**				
Distance from endpoint to obstacle	Street	3	0	1
	Oil/gas well	3	22.58	0
	Transmission line	3	35.04	0
	Highway	3	44.62	0
	Log step length (null model)	2	52.53	0
**E**				
Land cover at endpoint	CRP	3	0	1
Distance from endpoint to obstacle	Street	3	12.16	0
Land cover along step	CRP	3	15.08	0
Obstacle crossing	Log step length (null model)	2	64.69	0

*Note*: In addition to the eponymous variable, all models include log step length to account for bias towards short steps. The model with only log step length functions as a null model for this analysis. Models are ranked among each suite (Table 1A–D) and in an ensemble comparing the best model from each suite (E).

Abbreviation: CRP, Conservation Reserve Program grasslands.

^a^ Suites indicate groups of similar models.

^b^ Model names indicate the land cover/obstacle type used to construct the model.

^c^ Number of parameters in the model.

^d^ Number of Akaike Information Criterion units (corrected for small sample sizes) between the top and current model.

^e^ Model weight.

The four models in the distance from endpoint to obstacle suite all had high *β* estimates (0.13–0.34) compared to the top model (0.07), but only one (distance from endpoint to street, ΔAIC_c_ = 12.16) was within 30 ΔAIC_c_ units of the top model. The contrast between high *β* estimates and low model rankings for most models in the distance from endpoint to obstacle suite may be partially due to the distribution of these obstacles on the landscape. The distribution of oil and gas wells, which had the highest beta estimate of any covariate in the distance from endpoint to obstacle suite, was fairly uniform within each study area, and most used and available steps had similar distances from their endpoints to the nearest oil/gas well (Appendix S4: Figure S4). Highways and transmission lines are uncommon in the study area, and most used and available steps were equally unlikely to cross a highway or transmission line (Appendix S4: Figure S5).

## 4 DISCUSSION

In contrast to our prediction, we found that lesser prairie‐chicken dispersal after translocation is almost universal, with 98% of all translocated birds that survived from their spring release date until June undergoing a dispersal movement after release. The frequency of dispersal movements was similarly high between our two release areas (Colorado: 100%, Kansas: 94%), despite ecological differences between the sites. The lack of variation in dispersal between these two release areas, which varied in habitat types and concentration of anthropogenic features, seems to instead indicate that lesser prairie‐chicken dispersal after translocation is occurring in response to some other intrinsic or extrinsic stimulus. Gouar et al. (2008) suggested that animals might be dispersing after translocation in search of conspecifics, but the presence of leks in both release areas was unable to reduce the rate of post‐translocation dispersal. Other possible stimuli for dispersal include stress associated with release in a new area (Berger‐Tal et al., 2020), homing behaviors (Bell et al., 2010), and exploratory movements (Kemink & Kesler, 2013). Kemink and Kesler (2013) ascribed exploratory behaviors to translocated greater prairie‐chickens (*Tympanuchus cupido*) based on looping patterns observed in their dispersal movements. Similar looping patterns (Figure 4) suggest that exploratory behaviors are likely at least a partial motivator for the dispersal movements observed in our study.

Our findings support the hypothesis that lesser prairie‐chickens utilize grasslands during their dispersal movements, particularly supporting the role of CRP grassland in the Sand Sagebrush Prairie Ecoregion. CRP grassland is known to frequently contain habitat for lesser prairie‐chickens, especially during nesting and roosting, and provides important benefits for lesser prairie‐chicken persistence at a landscape scale (Hagen et al., 2020; Sullins et al., 2018; Tanner et al., 2021). Translocated lesser prairie‐chickens tended to make a single long‐distance movement per day, primarily at sunrise but also occasionally at sunset. During these movements, lesser prairie‐chickens selected for CRP grassland at the end of their dispersal steps and, contrary to our predictions, were tolerant of crossing obstacles such as transmission lines, streets, and highways as long as their end destination was distant from these obstacles. These results suggest a “stepping stone” method of transit for lesser prairie‐chickens during dispersal, where lesser prairie‐chickens make a single dispersal flight per day and then settle in patches of CRP grassland at the conclusion of these flights. The cover types that lesser prairie‐chickens cross during these flights have some relevance to their transit (CRP grassland along step, ΔAIC_c_ = 15.08), but the presence of CRP at the end of the step was the most important factor of lesser prairie‐chicken habitat selection during dispersal.

In contrast to their selection for CRP at the end of their dispersal steps, lesser prairie‐chickens did not select for native grassland during their dispersal movements. This is likely due to a lack of lesser prairie‐chicken habitat on native grassland in the Sand Sagebrush Prairie Ecoregion. Analysis of vegetation measurements from the Cimarron and Comanche National Grasslands as well as native rangelands suggests that native grasslands are not currently providing resources for lesser prairie‐chicken occupancy, including vegetation cover and appropriate plant communities (Berigan et al., 2022). Outside of our study area, however, native grassland does provide vegetation cover and plant communities amenable to lesser prairie‐chicken occupancy (Short‐Grass Prairie/CRP Mosaic Ecoregion, Kraft, 2016; Mixed‐Grass Prairie Ecoregion, Lautenbach, 2017; Sand Shinnery Oak Prairie Ecoregion, Schilder et al., 2022). It is likely that heterogenous lesser prairie‐chicken habitats, including both CRP and native grassland, can facilitate lesser prairie‐chicken dispersal movements provided that the grasslands have appropriate vegetation composition and structure for lesser prairie‐chicken occupancy (habitat requirements detailed in Hagen et al., 2004; Haukos & Zavaleta, 2016).

Our results demonstrate that patches of lesser prairie‐chicken habitat separated by 5 km or less should be accessible during a female lesser prairie‐chicken's average daily dispersal movement, and therefore facilitate female dispersal across the landscape. Populations inhabiting habitat patches that are connected by dispersal pathways may be more likely to persist through stochastic events (Rudnick et al., 2012). Extended droughts, for example, can cause local extinctions for lesser prairie‐chickens (Hagen et al., 2009; Ross et al., 2016b); landscape connectivity can facilitate recolonization of vacated patches when conditions improve (Garton et al., 2016). Our results suggest that landscape connectivity for lesser prairie‐chickens can be facilitated by strategically restoring/conserving quality habitat (e.g., CRP) to ensure a landscape configuration that connects populations or facilitates colonization of unoccupied, available habitat. While considerable variation in daily dispersal length (3.75 ± 4.95 km) shows that lesser prairie‐chickens have the ability to cross gaps between grassland patches, lesser prairie‐chicken dispersal connectivity may be optimized by ensuring that quality grassland patches are accessible at the end of their daily dispersal steps. Strategically conserving grassland habitat to ensure connectivity across landscapes will provide the opportunity for lesser prairie‐chickens to recolonize patches following local extinctions, therefore increasing the likelihood of lesser prairie‐chicken population persistence and increasing the availability of resources.

Dispersal movements that occur after lesser prairie‐chicken translocations have implications for the success of translocation efforts due to both direct lesser prairie‐chicken mortality and the diffusion of birds throughout the landscape. During our study, we observed lesser prairie‐chicken dispersal movements averaging 103 ± 73 km in length for males and 175 ± 108 km in length for females following translocation. Observed movements were >5 times longer than those measured in native populations (Earl et al., 2016) and likely contributed to mortality for translocated birds. Of all released birds, 13.1% died (*n* = 54) and 9.7% went missing (*n* = 40) within the first 2 weeks after release, indicating potentially substantial mortality associated with the act of translocation, adjustment to the new environment, and dispersal movement after translocation (Teige et al., 2023). High initial mortality rates are not unprecedented among prairie grouse translocations; Mathews et al. (2022) found similar rates of mortality (0.992 daily survival) within the first 40 days after Columbian sharp‐tailed grouse (*Tympanuchus phasianellus columbianus*) translocation.

At the end of their dispersal movements, translocated lesser prairie‐chickens settled at appreciable distances from their release sites (males: 13 ± 21 km, range = 0.5–64 km; females: 23 ± 20 km, range = 0.7–69 km), with 69% of birds settling >5 km from the release site. Males formed a series of small leks averaging 39.2 km ± 2 (SE; range = 0.0–77.0 km) from the nearest release site (maximum of 21 leks averaging 5.8 birds each in 2020; Teige et al., 2023). Although most long‐distance movements from both sexes occurred at sunrise, when lekking activity is strongest, the presence of leks did not appear to influence female selection during their dispersal movements. Females frequently settled in areas relatively distant from the nearest known lek (8.85 ± 8.82 km) and nested in areas that were similarly distant from leks (7.9 ± 8.9 km).

In circumstances where translocated animals undergo high rates of dispersal away from the release site, managers typically attempt to compensate by either (1) reducing dispersal rates, (2) translocating more individuals to compensate for high dispersal rates, or (3) choosing release sites that are less prone to dispersal (Armstrong et al., 2013). Prairie and sage grouse translocations have often focused on reducing dispersal rates using techniques such as brood translocation, which can result in lower dispersal propensity in exchange for a much higher cost per bird translocated (Huschle & Toepfer, 2020; Meyerpeter et al., 2021). While such a technique may be feasible for lesser prairie‐chickens, the larger cost associated with such an effort would necessitate translocating small numbers of individuals, which has traditionally been a risk factor for failure of prairie grouse translocations (Snyder et al., 1999). In lieu of a technique that can effectively reduce dispersal propensity at a reasonable cost, we suggest that lesser prairie‐chicken translocation should not focus on single‐site restoration or management. Richardson et al. (2015) suggest that translocations of species with a high dispersal propensity should instead select release sites based on landscape‐scale suitability for species reintroduction, with the expectation that animals will disperse away from the release site and settle in nearby areas. We posit that lesser prairie‐chicken translocation efforts should also focus on landscape‐scale habitat suitability, with the understanding that translocation acts as an ecoregion‐supplementation effort rather than a site‐specific restoration technique.

Diffusion of birds across the landscape resulted in issues with small population effects in the years following the translocation, with 122 males at 21 active leks in 2020 declining to 48 males at 10 active leks by 2022 (Teige et al., 2023). A small established population size following translocation could result in reintroduction failure in newly introduced populations due to Allee effects (Armstrong & Wittmer, 2011) or stochastic population fluctuations (Shaffer, 1981). The sheer number of birds released (411 birds in 2016–2019) was intended to serve as some insulation against small population effects for our translocation; however, widespread dispersal throughout the release area largely negated this effect. In addition to translocation serving as a regional population supplementation tool rather than a single‐site restoration technique, translocations will require large numbers of birds to overcome the high post‐translocation mortality and Allee effects associated with the dispersal of translocated birds across the landscape.

## AUTHOR CONTRIBUTIONS

**Liam A. Berigan:** Formal analysis (lead); investigation (equal); writing – original draft (lead); writing – review and editing (equal). **Carly S. H. Aulicky:** Investigation (equal); writing – review and editing (equal). **Elisabeth C. Teige:** Investigation (equal); writing – review and editing (equal). **Daniel S. Sullins:** Investigation (equal); writing – review and editing (equal). **Kent A. Fricke:** Conceptualization (equal); formal analysis (equal); writing – review and editing (equal). **Jonathan H. Reitz:** Conceptualization (equal); funding acquisition (equal); investigation (equal); writing – review and editing (equal). **Liza G. Rossi:** Conceptualization (equal); funding acquisition (equal); writing – review and editing (equal). **Kraig A. Schultz:** Conceptualization (equal); investigation (equal); writing – review and editing (equal). **Mindy B. Rice:** Supervision (supporting); writing – review and editing (equal). **Evan Tanner:** Resources (equal); writing – review and editing (equal). **Samuel D. Fuhlendorf:** Resources (equal); writing – review and editing (equal). **David A. Haukos:** Conceptualization (equal); resources (equal); supervision (lead); writing – original draft (supporting); writing – review and editing (equal).

## FUNDING INFORMATION

This project was funded by Federal Aid in Wildlife Restoration grant W‐98‐R‐1 through the Kansas Department of Wildlife and Parks and Colorado Parks and Wildlife, U.S. Geological Survey Kansas Cooperative Fish and Wildlife Research Unit, and the Division of Biology at Kansas State University. Funding was also provided through federal assistance via the State Wildlife Grants (CFDA Program No. 15.634) as well as state funding from Colorado Parks and Wildlife.

## CONFLICT OF INTEREST STATEMENT

The authors declare that they have no competing interests.

## Supporting information


Data S1

## Data Availability

Data are not publicly available for this study. The lesser prairie‐chicken is a threatened and endangered species under the United States Endangered Species Act, and any location data from this species are sensitive. Data may be provided to individual researchers upon reasonable request (contact: Kansas Cooperative Fish and Wildlife Research Unit, kscfwru@ksu.edu).

## References

[ece310871-bib-0001] Acevedo, C. J. , Koprowski, J. L. , Cavalcant, C. , Harding, L. , & Heffelfinger, J. R. (2023). The efficacy of translocation as a tool to augment populations of Gambel's quail. Journal of Wildlife Management, 87, e22359.

[ece310871-bib-0002] Armstrong, D. P. , McArthur, N. , Govella, S. , Morgan, K. , Johnston, R. , Gorman, N. , Pike, R. , & Richard, Y. (2013). Using radio‐tracking data to predict post‐release establishment in reintroductions to habitat fragments. Biological Conservation, 168, 152–160.

[ece310871-bib-0003] Armstrong, D. P. , & Wittmer, H. U. (2011). Incorporating Allee effects into reintroduction strategies. Ecological Research, 26, 687–695.

[ece310871-bib-0004] Bell, B. D. , Bishop, P. J. , Germano, J. M. , & Wellington, P. O. (2010). Lessons learned from a series of translocations of the archaic Hamilton's frog and Maud Island frog in central New Zealand. In P. S. Soorae (Ed.), Global re‐introduction perspectives: Additional case‐studies from around the globe (pp. 81–87). IUCN/SSC Re‐introduction Specialist Group & Environment Agency‐Abu Dhabi. https://iucn‐ctsg.org/wp‐content/uploads/publications/20_2010_Hamiltons_Maud_Island_Frog_New%20Zealand.pdf

[ece310871-bib-0005] Berger‐Tal, O. , Blumstein, D. T. , & Swaisgood, R. R. (2020). Conservation translocations: A review of common difficulties and promising directions. Animal Conservation, 23, 121–131.

[ece310871-bib-0006] Berigan, L. A. , Aulicky, C. S. H. , Teige, E. C. , Sullins, D. S. , Haukos, D. A. , Fricke, K. A. , Reitz, J. H. , Rossi, L. G. , Schultz, K. A. , & Ricketts, A. M. (2022). Availability of lesser prairie‐chicken nesting habitat impairs restoration success. Wildlife Society Bulletin, 46, e1379.

[ece310871-bib-0007] Boal, C. W. , & Haukos, D. A. (2016). The lesser prairie‐chicken: A brief introduction to the grouse of the southern Great Plains. In C. W. Boal & D. A. Haukos (Eds.), Ecology and conservation of lesser prairie‐chickens (pp. 20–33). CRC Press. https://pubs.er.usgs.gov/publication/70191988

[ece310871-bib-0008] Boryan, C. , Yang, Z. , Mueller, R. , & Craig, M. (2011). Monitoring US agriculture: The US Department of Agriculture, National Agricultural Statistics Service, Cropland Data Layer Program. Geocarto International, 26, 341–358.

[ece310871-bib-0009] Brooks, M. E. , Kristensen, K. , van Benthem, K. J. , Magnusson, A. , Berg, C. W. , Nielsen, A. , Skaug, H. J. , Maechler, M. , & Bolker, B. M. (2017). glmmTMB balances speed and flexibility among packages for zero‐inflated generalized linear mixed modeling. The R Journal, 9, 378–400.

[ece310871-bib-0010] Burnham, K. P. , & Anderson, D. R. (1998). Model selection and multimodel inference. Springer.

[ece310871-bib-0011] Calenge, C. (2006). The package “adehabitat” for the R software: A tool for the analysis of space and habitat use by animals. Ecological Modelling, 197, 516–519.

[ece310871-bib-0012] Coates, P. S. , Stiver, S. J. , & Delehanty, D. J. (2006). Using sharp‐tailed grouse movement patterns to guide release‐site selection. Wildlife Society Bulletin, 34, 1376–1382.

[ece310871-bib-0013] Colorado Oil and Gas Conservation Commission . (2020). Well surface location data . https://cogcc.state.co.us/data2.html#/downloads

[ece310871-bib-0014] De Milliano, J. , Di Stefano, J. , Courtney, P. , Temple‐Smith, P. , & Coulson, G. (2016). Soft‐release versus hard‐release for reintroduction of an endangered species: An experimental comparison using eastern barred bandicoots (*Perameles gunnii*). Wildlife Research, 43, 1–12.

[ece310871-bib-0015] Earl, J. E. , Fuhlendorf, S. D. , Haukos, D. , Tanner, A. M. , Elmore, D. , & Carleton, S. A. (2016). Characteristics of lesser prairie‐chicken (*Tympanuchus pallidicinctus*) long‐distance movements across their distribution. Ecosphere, 7, e01441.

[ece310871-bib-0016] Epps, C. W. , Palsbøll, P. J. , Wehausen, J. D. , Roderick, G. K. , Ramey, R. R. , & McCullough, D. R. (2005). Highways block gene flow and cause a rapid decline in genetic diversity of desert bighorn sheep: Highways reduce genetic diversity. Ecology Letters, 8, 1029–1038.

[ece310871-bib-0017] Fortin, D. , Beyer, H. L. , Boyce, M. S. , Smith, D. W. , Duchesne, T. , & Mao, J. S. (2005). Wolves influence elk movements: Behavior shapes a trophic cascade in Yellowstone National Park. Ecology, 86, 1320–1330.

[ece310871-bib-0018] Fuhlendorf, S. D. , Woodward, A. J. , Leslie, D. M. , & Shackford, J. S. (2002). Multi‐scale effects of habitat loss and fragmentation on lesser prairie‐chicken populations of the US Southern Great Plains. Landscape Ecology, 17, 617–628.

[ece310871-bib-0019] Garton, E. , Hagen, C. , Beauprez, G. M. , Kyle, S. C. , Pitman, J. C. , Schoeling, D. S. , & Pelt, W. E. (2016). Population dynamics of the lesser prairie‐chicken. In C. W. Boal & D. A. Haukos (Eds.), Ecology and management of lesser prairie‐chickens (pp. 49–76). CRC Press. https://pubs.er.usgs.gov/publication/70191988

[ece310871-bib-0020] Gil‐Tena, A. , Brotons, L. , Fortin, M.‐J. , Burel, F. , & Saura, S. (2013). Assessing the role of landscape connectivity in recent woodpecker range expansion in Mediterranean Europe: Forest management implications. European Journal of Forest Research, 132, 181–194.

[ece310871-bib-0021] Gouar, P. L. , Robert, A. , Choisy, J.‐P. , Henriquet, S. , Lecuyer, P. , Tessier, C. , & Sarrazin, F. (2008). Roles of survival and dispersal in reintroduction success of Griffon Vulture (*Gyps fulvus*). Ecological Applications, 18(4), 859–872. 18536248 10.1890/07-0854.1

[ece310871-bib-0022] Griffith, B. , Scott, J. M. , Carpenter, J. W. , & Reed, C. (1989). Translocation as a species conservation tool: Status and strategy. Science, 245, 477–480. 17750257 10.1126/science.245.4917.477

[ece310871-bib-0023] Haddad, N. M. , Brudvig, L. A. , Clobert, J. , Davies, K. F. , Gonzalez, A. , Holt, R. D. , Lovejoy, T. E. , Sexton, J. O. , Austin, M. P. , Collins, C. D. , Cook, W. M. , Damschen, E. I. , Ewers, R. M. , Foster, B. L. , Jenkins, C. N. , King, A. J. , Laurance, W. F. , Levey, D. J. , Margules, C. R. , … Townshend, J. R. (2015). Habitat fragmentation and its lasting impact on Earth's ecosystems. Science Advances, 1, e1500052. 26601154 10.1126/sciadv.1500052 PMC4643828

[ece310871-bib-0024] Hagen, C. , Carlisle, J. , Hornsby, F. , Houts, M. , McDonald, L. , & Pavlacky, D., Jr. (2020). Multiscale occupancy of the lesser prairie‐chicken: The role of private lands in conservation of an imperiled bird. Avian Conservation and Ecology, 15(2), 17.

[ece310871-bib-0025] Hagen, C. A. , Jamison, B. E. , Giesen, K. M. , & Riley, T. Z. (2004). Guidelines for managing lesser prairie‐chicken populations and their habitats. Wildlife Society Bulletin, 32, 69–82. 32327860 10.2193/0091-7648(2004)32[69:GFMLPP]2.0.CO;2 PMC7169742

[ece310871-bib-0026] Hagen, C. A. , Sandercock, B. K. , Pitman, J. C. , Robel, R. J. , & Applegate, R. D. (2009). Spatial variation in lesser prairie‐chicken demography: A sensitivity analysis of population dynamics and management alternatives. Journal of Wildlife Management, 73, 1325–1332.

[ece310871-bib-0027] Hamerstrom, F. N., Jr. , & Hamerstrom, F. (1951). Mobility of the sharp‐tailed grouse in relation to its ecology and distribution. American Midland Naturalist, 46(1), 174–226.

[ece310871-bib-0028] Haukos, D. A. , & Zavaleta, J. (2016). Habitat. In D. A. Haukos & C. W. Boal (Eds.), Ecology and management of lesser Prairie‐Chickens (pp. 49–76). CRC Press.

[ece310871-bib-0029] High Plains Regional Climate Center . (2023). Station level data . https://hprcc.unl.edu/datasets.php?set=CountyData

[ece310871-bib-0030] Huschle, G. , & Toepfer, J. E. (2020). Trends in a greater prairie‐chicken population established by translocation in North Dakota. Prairie Naturalist, 52(2), 76–79.

[ece310871-bib-0031] IUCN/SSC . (2013). Guidelines for reintroductions and other conservation translocations. Version 1.0. IUCN Species Survival Commission.

[ece310871-bib-0032] Kansas Geological Survey . (2020). Well location data . http://www.kgs.ku.edu/PRS/petroDB.html#ASCII

[ece310871-bib-0033] Kemink, K. M. , & Kesler, D. C. (2013). Using movement ecology to inform translocation efforts: A case study with an endangered lekking bird species. Animal Conservation, 16, 449–457.

[ece310871-bib-0034] Kraft, J. D. (2016). Vegetation characteristics and lesser prairie chicken responses to land cover types and grazing management in western Kansas (Thesis). Kansas State University. http://hdl.handle.net/2097/34550

[ece310871-bib-0035] Lautenbach, J. D. (2017). The role of fire, microclimate, and vegetation in lesser prairie‐chicken habitat selection. Thesis, Kansas State University. http://hdl.handle.net/2097/35395

[ece310871-bib-0036] Lautenbach, J. M. , Haukos, D. A. , Sullins, D. S. , Hagen, C. A. , Lautenbach, J. D. , Pitman, J. C. , Plumb, R. T. , Robinson, S. G. , & Kraft, J. D. (2019). Factors influencing nesting ecology of lesser prairie‐chickens. Journal of Wildlife Management, 83, 205–215.

[ece310871-bib-0037] Mathews, S. R. , Coates, P. S. , Prochazka, B. G. , Espinosa, S. P. , & Delehanty, D. J. (2022). Survival of translocated Columbian sharp‐tailed grouse: Recognizing trends in post‐release mortality to improve reintroductions. Avian Conservation and Ecology, 17(2), 28.

[ece310871-bib-0038] McDonald, L. , Beauprez, G. , Gardner, G. , Griswold, J. , Hagen, C. , Hornsby, F. , Klute, D. , Kyle, S. , Pitman, J. , & Rintz, T. (2014). Range‐wide population size of the lesser prairie‐chicken: 2012 and 2013. Wildlife Society Bulletin, 38, 536–546.

[ece310871-bib-0039] Meyerpeter, M. B. , Lazenby, K. D. , Coates, P. S. , Ricca, M. A. , Mathews, S. R. , Gardner, S. C. , Dahlgren, D. K. , & Delehanty, D. J. (2021). Field methods for translocating female greater sage‐grouse (*Centrocercus urophasianus*) with their broods. Wildlife Society Bulletin, 45, 529–537.

[ece310871-bib-0040] Michelot, T. , Langrock, R. , & Patterson, T. A. (2016). moveHMM: An R package for the statistical modelling of animal movement data using hidden Markov models. Methods in Ecology and Evolution, 7, 1308–1315.

[ece310871-bib-0041] Muff, S. , Signer, J. , & Fieberg, J. (2020). Accounting for individual‐specific variation in habitat‐selection studies: Efficient estimation of mixed‐effects models using Bayesian or frequentist computation. Journal of Animal Ecology, 89, 80–92. 31454066 10.1111/1365-2656.13087

[ece310871-bib-0042] Nilsson, K. , Pearson, D. , Paxman, M. , Desmond, A. , Kennington, J. , Byrne, M. , & Ottewell, K. (2023). Translocations restore a population of a threatened rock‐wallaby and bolster its genetic diversity. Conservation Genetics, 24, 547–561.

[ece310871-bib-0043] Oklahoma Corporation Commission . (2020). Well completion list . https://oklahoma.gov/occ/divisions/oil‐gas/oil‐gas‐data.html

[ece310871-bib-0044] Peterson, J. M. , Earl, J. E. , Fuhlendorf, S. D. , Elmore, R. D. , Haukos, D. A. , Tanner, A. M. , & Carleton, S. A. (2020). Estimating response distances of lesser prairie‐chickens to anthropogenic features during long‐distance movements. Ecosphere, 11, e03202.

[ece310871-bib-0045] Pulliam, H. (1988). Sources, sinks and population regulation. American Naturalist, 132, 652–661.

[ece310871-bib-0046] R Core Team . (2023). R: A language and environment for statistical computing. R Foundation for Statistical Computing. http://www.r‐project.org

[ece310871-bib-0047] Reese, K. P. , & Connelly, J. W. (1997). Translocations of sage grouse Centrocercus urophasianus in North America. Wildlife Biology, 3, 235–241.

[ece310871-bib-0048] Richardson, K. M. , Doerr, V. , Ebrahimi, M. , Lovegrove, T. G. , & Parker, K. A. (2015). Considering dispersal in reintroduction and restoration planning. In D. P. Armstrong , M. Hayward , D. Moro , & P. Seddon (Eds.), Advances in reintroduction biology of Australian and New Zealand fauna (pp. 59–72). CSIRO Publishing.

[ece310871-bib-0049] Rodgers, R. D. (2016). A history of lesser prairie‐chickens. In D. A. Haukos & C. W. Boal (Eds.), Ecology and conservation of lesser prairie‐chickens (pp. 15–38). CRC Press.

[ece310871-bib-0050] Ross, B. E. , Haukos, D. , Hagen, C. , & Pitman, J. (2016b). The relative contribution of climate to changes in lesser prairie‐chicken abundance. Ecosphere, 7, e01323.

[ece310871-bib-0051] Ross, B. E. , Haukos, D. A. , Hagen, C. A. , & Pitman, J. C. (2016a). Landscape composition creates a threshold influencing lesser prairie‐chicken population resilience to extreme drought. Global Ecology and Conservation, 6, 179–188.

[ece310871-bib-0052] Rudnick, D. , Ryan, S. J. , Beier, P. , Cushman, S. A. , Dieffenbach, F. , Epps, C. , Gerber, L. R. , Hartter, J. N. , Jenness, J. S. , Kintsch, J. , Merenlender, A. M. , Perkl, R. M. , Perziosi, D. V. , & Trombulack, S. C. (2012). The role of landscape connectivity in planning and implementing conservation and restoration priorities. Issues in Ecology, 16, 1–20.

[ece310871-bib-0053] Sarremejane, R. , Truchy, A. , McKie, B. G. , Mykrä, H. , Johnson, R. K. , Huusko, A. , Sponseller, R. A. , & Muotka, T. (2021). Stochastic processes and ecological connectivity drive stream invertebrate community responses to short‐term drought. Journal of Animal Ecology, 90, 886–898. 33368270 10.1111/1365-2656.13417

[ece310871-bib-0054] Schilder, L. , Heintzman, L. , McIntyre, N. , Harryman, S. , Hagen, C. , Martin, R. , Boal, C. , & Grisham, B. (2022). Structural and functional landscape connectivity for lesser prairie‐chickens in the Sand Shinnery Oak Prairie Ecoregion. Journal of Wildlife Management, 86, e22146.

[ece310871-bib-0055] Shaffer, M. L. (1981). Minimum population sizes for species conservation. Bioscience, 31, 131–134.

[ece310871-bib-0056] Signer, J. , Fieberg, J. , & Avgar, T. (2019). Animal movement tools (amt): R package for managing tracking data and conducting habitat selection analyses. Ecology and Evolution, 9, 880–890. 30766677 10.1002/ece3.4823 PMC6362447

[ece310871-bib-0057] Smith, M. M. , Gilbert, J. H. , Olson, E. R. , Scribner, K. T. , Van Deelen, T. R. , Van Stappen, J. F. , Williams, B. W. , Woodford, J. E. , & Pauli, J. N. (2021). A recovery network leads to the natural recolonization of an archipelago and a potential trailing edge refuge. Ecological Applications, 31, e02416. 34278627 10.1002/eap.2416

[ece310871-bib-0058] Snyder, J. W. , Pelren, E. C. , & Crawford, J. A. (1999). Translocation histories of prairie grouse in the United States. Wildlife Society Bulletin, 27, 428–432.

[ece310871-bib-0059] Solomon, M. J. (2022). Evaluating habitat suitability for lesser prairie‐chicken conservation in the mixed‐grass prairie ecoregion (Thesis). Montana State University. wildlifehabitatecologylab.com/uploads/1/1/9/8/119890489/solomon_thesis_final.pdf

[ece310871-bib-0060] Spencer, D. , Haukos, D. , Hagen, C. , Daniels, M. , & Goodin, D. (2017). Conservation reserve program mitigates grassland loss in the lesser prairie‐chicken range of Kansas. Global Ecology and Conservation, 9, 21–38.

[ece310871-bib-0061] Stubbs, M. (2014). Conservation reserve program (CRP): Status and issues. Library of Congress, Congressional Research Service. Report No.: R42783. https://crsreports.congress.gov/product/pdf/R/R42783

[ece310871-bib-0062] Sullins, D. S. , Kraft, J. D. , Haukos, D. A. , Robinson, S. G. , Reitz, J. H. , Plumb, R. T. , Lautenbach, J. M. , Lautenbach, J. D. , Sandercock, B. K. , & Hagen, C. A. (2018). Demographic consequences of Conservation Reserve Program grasslands for lesser prairie‐chickens. Journal of Wildlife Management, 82, 1617–1632.

[ece310871-bib-0063] Tanner, E. P. , Tanner, A. M. , Fuhlendorf, S. D. , Elmore, R. D. , Davis, C. A. , & Polo, J. A. (2021). Land enrolled in the Conservation Reserve Program supports roosting ecology of the lesser prairie‐chicken. Global Ecology and Conservation, 32, e01916.

[ece310871-bib-0064] Taylor, P. D. , Fahrig, L. , Henein, K. , & Merriam, G. (1993). Connectivity is a vital element of landscape structure. Oikos, 68, 571–573.

[ece310871-bib-0065] Teige, E. C. , Berigan, L. A. , Aulicky, C. S. H. , Reitz, J. H. , Haukos, D. A. , Sullins, D. S. , Fricke, K. A. , Schultz, K. A. , & Rossi, L. G. (2023). Assessment of lesser prairie‐chicken translocation through survival and lek surveys. Wildlife Society Bulletin, 47, e1493.

[ece310871-bib-0066] Thurfjell, H. , Ciuti, S. , & Boyce, M. S. (2014). Applications of step‐selection functions in ecology and conservation. Movement Ecology, 2, 1–12. 25520815 10.1186/2051-3933-2-4 PMC4267544

[ece310871-bib-0067] U.S. Census Bureau . (2015). 2010 TIGER/Line® Shapefiles . https://www.census.gov/geographies/mapping‐files/time‐series/geo/tiger‐line‐file.html

[ece310871-bib-0068] Vogel, J. A. (2015). An unexpected journey: Greater prairie‐chicken travels nearly 4000 km after translocation to Iowa. American Midland Naturalist, 174, 343–349.

[ece310871-bib-0069] Westemeier, R. L. , Brawn, J. D. , Simpson, S. A. , Esker, T. L. , Jansen, R. W. , Walk, J. W. , Kershner, E. L. , Bouzat, J. L. , & Paige, K. N. (1998). Tracking the long‐term decline and recovery of an isolated population. Science, 282, 1695–1698. 9831558 10.1126/science.282.5394.1695

[ece310871-bib-0070] Yoder, J. M. , Marschall, E. A. , & Swanson, D. A. (2004). The cost of dispersal: Predation as a function of movement and site familiarity in ruffed grouse. Behavioral Ecology, 15, 469–476.

