# Supplementary material for: Lesser prairie‐chicken dispersal after translocation: Implications for restoration and population connectivity
Source: Ecol Evol. 2024 Jan 31;14(2):e10871. doi: 10.1002/ece3.10871 (PMC10828740; doi:10.1002/ece3.10871)
Supplement: Supplementary file 1 — Data S1 [file ECE3-14-e10871-s001.zip › ece310871-sup-0006-Supinfo.docx]

**Appendix S1. Additional figure S1 illustrating lesser prairie-chicken ecoregions and respective population trends**

Figure S1. Lesser prairie-chicken (*Tympanuchus pallidicinctus*) ecoregions sensu McDonald et al. (2014), with population trends from Nasman et al. (2022). Error bars represent 90% confidence intervals. Lesser prairie-chicken surveys were not conducted in 2019, and error bars could not be estimated for the Sand Sagebrush Prairie Ecoregion in 2020 due to low population size.

Nasman, K., T. Rintz, G. DiDonato, and F. Kulzer. 2022. Range-wide population size of the lesser prairie-chicken: 2012 to 2022. Western EcoSystems Technology, Inc., Fort Collins, Colorado. https://wafwa.org/wp-content/uploads/2022/11/LPC_RangeWidePopSize2012-2022.pdf.

**Appendix S2. Historical accounts of previous lesser prairie-chicken translocation efforts by Colorado Parks and Wildlife and Kansas Department of Wildlife and Parks.**

Colorado Parks and Wildlife and Kansas Department of Wildlife and Parks had conducted 4 lesser prairie-chicken translocation projects prior to the 2016–2019 translocation (Table A1). The first project took place in 1968 when 27 lesser prairie-chickens (13 males, 14 females) were transplanted from Kansas to the Sand Creek Drainage, and released 7 miles south of Hugo, Colorado. The aim of the translocation was to establish a new lesser prairie-chicken population in the Sand Creek Drainage. However, the effort failed as no leks were established at the release site. There is evidence that these birds may have undergone dispersal after translocation, leading to the establishment of a population of lesser prairie-chickens approximately 18 miles to the southeast of the release site, in an area with a broader expanse of quality habitat. One of the released birds was hit on a road 12 miles to the southeast of the release site, 1 year after the release. In the mid-1970s, landowners near the Cheyenne/Lincoln County border, 18 miles SE of the release site, started reporting occasional lesser prairie-chicken sightings. In 1998, Colorado Division of Wildlife found active leks in the area. The western Cheyenne County population of lesser prairie-chickens may have originated from a lek complex in Kiowa County, but the Hugo release site was closer to the western Cheyenne County leks than the Kiowa County leks.

In 1972 and 1975, 53 lesser prairie-chickens (28 males, 25 females) were translocated from Meade County, Kansas, to the U.S. Department of Agriculture (USDA)-Forest Service Sand Arroyo pastures in central Baca County, Colorado. The Sand Arroyo is currently a ~17,000 acre “island” of privately owned sand sagebrush rangeland, USDA-Forest Service grassland, and Conservation Reserve Program (CRP) grassland surrounded by heavily grazed shortgrass and farm ground. In 1975, agency biologists found the first post-Dust Bowl lesser prairie-chicken lek on the Sand Arroyo. A lesser prairie-chicken population persisted on the Sand Arroyo from 1975 to 1995, with a high count of 28 males on 2 leks in 1981. Since 1995, no leks have been observed on the Sand Arroyo.

A third translocation project took place in 1972 and 1975 to enhance genetic diversity within the Baca County, Colorado, population. A total of 31 males and 13 females were released over 2 years at release sites throughout the Baca County lesser prairie-chicken range. The population in Baca County increased steadily and significantly from 1972 until 1989, but it is unclear whether the supplementary releases contributed to the increase. It is difficult to evaluate the success of this translocation effort on the genetic diversity of the Baca County lesser prairie-chicken population.

The largest lesser prairie-chicken translocation prior to 2016 was to the Pueblo Railroad Transportation Test Center, near Pueblo, Colorado. This 32,000 acre site is well outside of the known range for lesser prairie-chickens, but consists of sand sagebrush rangeland similar to that seen within the Sand Sagebrush Prairie Ecoregion. Relatively small numbers of lesser prairie-chickens were transplanted from Colorado and Kansas in 1988, 1989, 1991, and 1993, with no more than 8 hens released in any given year. Translocation effort increased in 1994 with the release of 23 females and 26 males. In 1993 and 1994, very-high-frequency radio transmitters were attached to 23 birds to measure the success of the translocation. The fates of those 23 birds are as follows:

- 1 died at the time of release
- 11 were killed by coyotes and raptors
- 3 died of unknown causes
- 1 successfully raised a brood of 12, and then went missing
- 1 bird slipped its transmitter
- 6 birds went missing (unable to detect signal)

One of the 1993 released birds was killed by a hunter in Kearney County, Kansas, more than 200 miles from the release site, relatively close to its lek of capture. This translocation effort was unsuccessful, with no lek establishment at the Pueblo Test Track site. It is unknown if the cause of failure was due to insufficient habitat quality, insufficient numbers of birds, dispersal of released birds, or a combination of all three.

Table S1. Known historic lesser prairie-chicken translocations efforts involving Colorado Parks and Wildlife and Kansas Department of Wildlife and Parks.

| **Project** | **Date** | **Source** | **Birds(n)** | **Males** | **Females** | **Release Site** |
| --- | --- | --- | --- | --- | --- | --- |
| Gun Club Project | Spring 1961 | Colorado | 8 | ? | ? | NE 1/4 S20-T34-R46 Campo Rod and Gun Club Wildlife Area |
| Hugo Project | March 1968 | Kansas | 21 | 13 | 8 | 7 miles south of Hugo |
|  | April 1968 | Kansas | 6 | 0 | 6 | 7 miles south of Hugo |
| Sand Arroyo Project | January 1972 | Kansas- Meade County | 30 | 15 | 15* | S18-T32-R45 Arroyo Allotment |
|  | February 1972 | Kansas- Meade County | 5 | 3 | 2 | S18-T32-R45 Arroyo Allotment |
|  | January 1975 | Kansas | 10 | 6 | 4 | S35-T31-R45 Vilas Grade Allotment |
|  | February 1975 | Kansas | 8 | 4 | 4 | S19/20-T32-R45 Arroyo Allotment |
| Pueblo Test Track Project | April 1988 | Colorado (Baca and Prowers) | 25 | 17 | 8 | Pueblo Test Track |
|  | 1989-1991 | Colorado (Baca- south of Cimarron) | 13 | 9 | 4 | Pueblo Test Track |
|  | April 1993 | Kansas | 28 | 19 | 7 | Pueblo Test Track |
|  | April 1994 | Kansas | 49 | 26 | 23 | Pueblo Test Track |
| Genetic Diversity Project | January 1972 | Kansas- Meade County | 13 | 13 | 0 | S27-T34-R44 Mt. Carmel Allotment** |
|  | January 1975 | Kansas | 8 | 8 | 0 | S22-T34-R44 Border of Schnaufers and West edge of Sunflower |
|  | February 1975 | Kansas | 10 | 4 | 6 | S27-T34-R45 Aubrey Trail Allotment- Same section as Aubrey Trail Lek |
|  | February 1975 | Kansas | 10 | 3 | 7 | S5-T35-46W Deweese Allotment |
|  | February 1975 | Kansas | 3 | 3 | 0 | S22-T34-R44 Border of Schnaufers and West edge of Sunflower |

*One female was released in poor condition following trapping.

**Males released on Mt Carmel Allotment due to having excess males available from the Sand Arroyo translocation project.

**Appendix S3. Differences in step selection results between male and female lesser prairie-chickens**.

Figure S2. Male and female beta estimates with 95% confidence intervals for all variables used as covariates in a step selection function (Fortin et al. 2005) used to determine how lesser prairie-chickens (*Tympanuchus pallidicinctus*) select for landscape features during dispersal after release in southeastern Colorado and southwestern Kansas in 2016–2019. Beta estimates are extracted from single variable models with *z*-scaled covariates. Positive estimates demonstrate selection for a variable, while negative estimates demonstrate avoidance. Note that selection for covariates in the “distance from endpoint to obstacle” category constitutes avoidance of the appropriate obstacle: e.g., lesser prairie-chickens select for steps whose endpoints are far from oil wells. Asterisks mark the most informative model in each suite, as determined using Akaike Information Criterion (corrected for small sample sizes). The absence of an asterisk in a suite indicates that the null model was the most informative model.

Tables S2A–S2E. Male model selection tables used to determine the effect of land cover and obstacles on lesser prairie-chicken (*Tympanuchus pallidicinctus*) step selection during dispersal after translocation to Morton, Kansas and Baca, Colorado in 2018–2019.

Table S2A

| **Suite^1^** | **Model^2^** | **K^3^** | **ΔAICc^4^** | ***w*_i_^5^** |
| --- | --- | --- | --- | --- |
| Land cover along step | Non-CRP grassland | 3 | 0 | 1 |
|  | Shrubland | 3 | 17.83 | 0 |
|  | CRP | 3 | 36.02 | 0 |
|  | Developed (open) | 3 | 41.86 | 0 |
|  | Log step length (null model) | 2 | 42.95 | 0 |

Table S2B

| **Suite^1^** | **Model^2^** | **K^3^** | **ΔAICc^4^** | ***w*_i_^5^** |
| --- | --- | --- | --- | --- |
| Land cover at endpoint | Shrubland | 3 | 0 | 0.85 |
|  | CRP | 3 | 4.44 | 0.09 |
|  | Non-CRP grassland | 3 | 5.55 | 0.05 |
|  | Developed (open) | 3 | 24.45 | 0 |
|  | Log step length (null model) | 2 | 26.34 | 0 |

Table S2C

| **Suite^1^** | **Model^2^** | **K^3^** | **ΔAICc^4^** | ***w*_i_^5^** |
| --- | --- | --- | --- | --- |
| Obstacle crossing | Log step length (null model) | 2 | 0 | 0.47 |
|  | Street | 3 | 1.29 | 0.25 |
|  | Highway | 3 | 1.65 | 0.21 |
|  | Transmission line | 3 | 3.83 | 0.07 |

Table S2D

| **Suite^1^** | **Model^2^** | **K^3^** | **ΔAICc^4^** | ***w*_i_^5^** |
| --- | --- | --- | --- | --- |
| Distance from endpoint to obstacle | Street | 3 | 0 | 1 |
|  | Oil/gas well | 3 | 15.43 | 0 |
|  | Transmission line | 3 | 19.43 | 0 |
|  | Highway | 3 | 26.31 | 0 |
|  | Log step length (null model) | 2 | 31.98 | 0 |

Table S2E

| **Suite^1^** | **Model^2^** | **K^3^** | **ΔAICc^4^** | ***w*_i_^5^** |
| --- | --- | --- | --- | --- |
| Land cover along step | Non-CRP grassland | 3 | 0 | 1 |
| Distance from endpoint to obstacle | Street | 3 | 10.97 | 0 |
| Land cover at endpoint | Shrubland | 3 | 16.61 | 0 |
| Obstacle crossing | Log step length (null model) | 2 | 42.95 | 0 |

Abbreviations: Conservation Reserve Program grasslands (CRP)

Notes: In addition to the eponymous variable, all models include log step length to account for bias towards short steps. The model with only log step length functions as a null model for this analysis. Models are ranked among each suite (Tables S2A–S2D) and in an ensemble comparing the best model from each suite (S2E).

Footnotes: ^1^ Suites indicate groups of similar models. ^2^ Model names indicate the land cover/obstacle type used to construct the model. ^3^ Number of parameters in the model. ^4^ Number of Akaike Information Criterion units (corrected for small sample sizes) between the top and current model. ^5^ Model weight.

Tables S3A–S3E. Female model selection tables used to determine the impact of land cover and obstacles on lesser prairie-chicken (*Tympanuchus pallidicinctus*) step selection during dispersal after translocation to Morton, Kansas and Baca, Colorado in 2018–2019.

Table S3A

| **Suite^1^** | **Model^2^** | **K^3^** | **ΔAICc^4^** | ***w*_i_^5^** |
| --- | --- | --- | --- | --- |
| Land cover along step | CRP | 3 | 0 | 1 |
|  | Non-CRP grassland | 3 | 26.73 | 0 |
|  | Shrubland | 3 | 28.95 | 0 |
|  | Developed (open) | 3 | 33.16 | 0 |
|  | Log step length (null model) | 2 | 36.04 | 0 |

Table S3B

| **Suite^1^** | **Model^2^** | **K^3^** | **ΔAICc^4^** | ***w*_i_^5^** |
| --- | --- | --- | --- | --- |
| Land cover at endpoint | CRP | 3 | 0 | 1 |
|  | Non-CRP grassland | 3 | 26.08 | 0 |
|  | Shrubland | 3 | 28.86 | 0 |
|  | Developed (open) | 3 | 34.40 | 0 |
|  | Log step length (null model) | 2 | 35.72 | 0 |

Table S3C

| **Suite^1^** | **Model^2^** | **K^3^** | **ΔAICc^4^** | ***w*_i_^5^** |
| --- | --- | --- | --- | --- |
| Obstacle crossing | Log step length (null model) | 2 | 0 | 0.58 |
|  | Transmission line | 3 | 1.89 | 0.23 |
|  | Street | 3 | 3.27 | 0.11 |
|  | Highway | 3 | 3.94 | 0.08 |

Table S3D

| **Suite^1^** | **Model^2^** | **K^3^** | **ΔAICc^4^** | ***w*_i_^5^** |
| --- | --- | --- | --- | --- |
| Distance from endpoint to obstacle | Street | 3 | 0 | 1 |
|  | Oil/gas well | 3 | 12.19 | 0 |
|  | Transmission line | 3 | 15.33 | 0 |
|  | Highway | 3 | 21.99 | 0 |
|  | Log step length (null model) | 2 | 22.26 | 0 |

Table S3E

| **Suite^1^** | **Model^2^** | **K^3^** | **ΔAICc^4^** | ***w*_i_^5^** |
| --- | --- | --- | --- | --- |
| Land cover along step | CRP | 3 | 0 | 0.54 |
| Land cover at endpoint | CRP | 3 | 0.33 | 0.46 |
| Distance from endpoint to obstacle | Street | 3 | 13.78 | 0 |
| Obstacle crossing | Log step length (null model) | 2 | 36.04 | 0 |

Abbreviations: Conservation Reserve Program grasslands (CRP)

Notes: In addition to the eponymous variable, all models include log step length to account for bias towards short steps. The model with only log step length functions as a null model for this analysis. Models are ranked among each suite (Tables S3A–S3D) and in an ensemble comparing the best model from each suite (S3E).

Footnotes: ^1^ Suites indicate groups of similar models. ^2^ Model names indicate the land cover/obstacle type used to construct the model. ^3^ Number of parameters in the model. ^4^ Number of Akaike Information Criterion units (corrected for small sample sizes) between the top and current model. ^5^ Model weight.

**Appendix S4. Additional figures S3–S5 describing distributions of nests and obstacles on the landscape in southwestern Kansas and southeastern Colorado, USA.**

Figure S3. Distribution of nests laid by translocated lesser prairie-chickens during 2017–2019 in southwestern Kansas and southeastern Colorado, USA, in relation to the locations of leks and their immediate surroundings (3.2 km radius). Almost all nests would fall within 3.2 km of a lek in a native population (Boal and Haukos 2016).

Figure S4. Distance from endpoint to oil/gas well for used/available steps in the step selection function, and distribution of active oil/gas wells in southeastern Colorado and southwestern Kansas. Translocated lesser prairie-chickens in 2017–2019 were released in areas where oil/gas wells were sparse (Comanche National Grassland) or ubiquitous (Cimarron National Grassland), which may have made this model less informative in predicting lesser prairie-chicken step selection.

Figure S5. Distribution of highways and transmission lines in southeastern Colorado and southwestern Kansas, and distance from endpoint to transmission line or highway for used/available steps in a step selection function examining the movements of lesser prairie-chickens translocated in 2018–2019. Distance from transmission line/highway to endpoint models may have been less informative because highways and transmission lines were distant from many portions of the study area.
