# Supplementary figures and images for: Lesser prairie‐chicken dispersal after translocation: Implications for restoration and population connectivity
Source: Ecol Evol. 2024 Jan 31;14(2):e10871. doi: 10.1002/ece3.10871 (PMC10828740; doi:10.1002/ece3.10871)

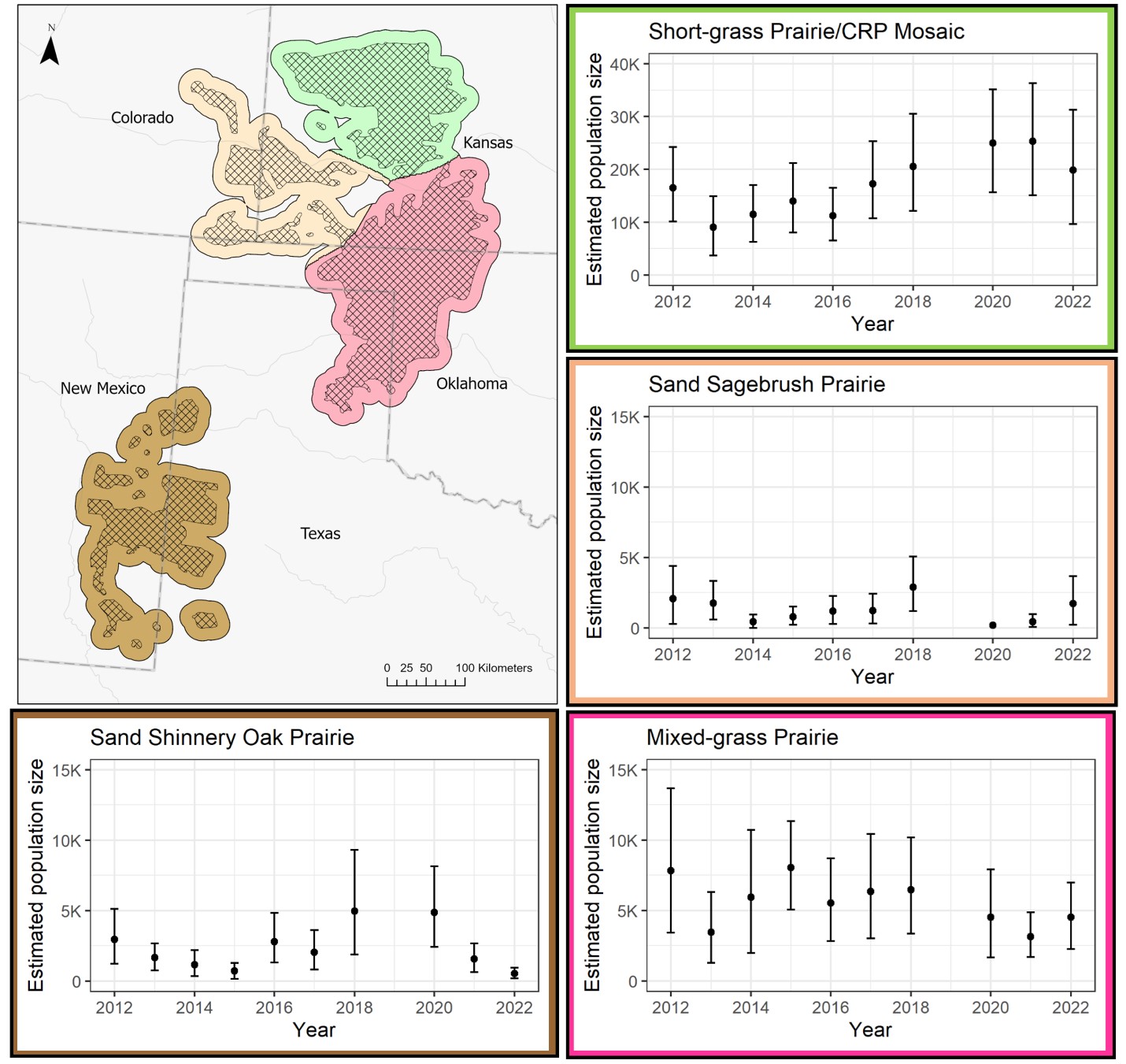

Supplement: Supplementary file 1 — Data S1 [file ECE3-14-e10871-s001.zip › ece310871-sup-0001-FigureS1.jpg]

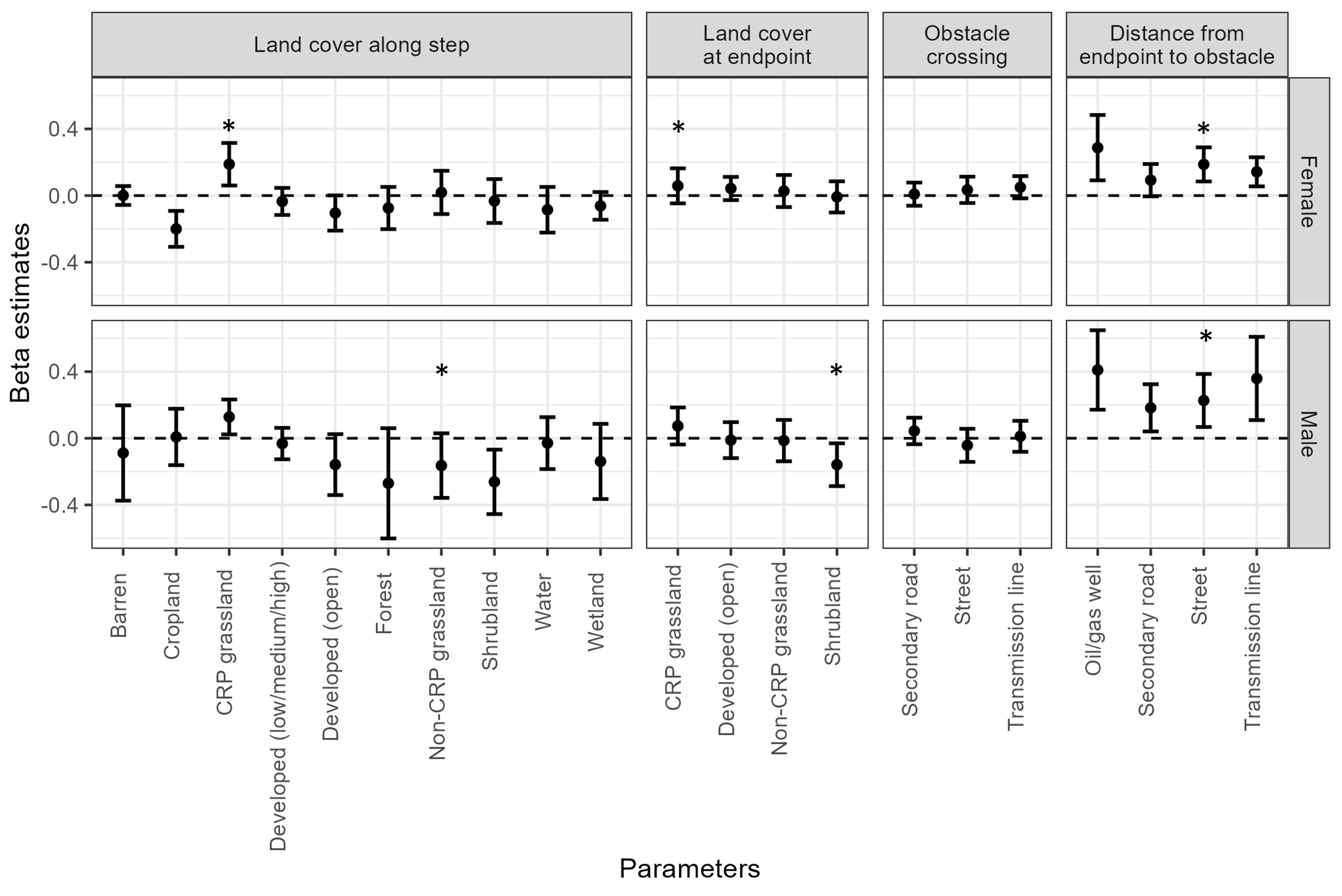

Supplement: Supplementary file 1 — Data S1 [file ECE3-14-e10871-s001.zip › ece310871-sup-0002-FigureS2.tif]

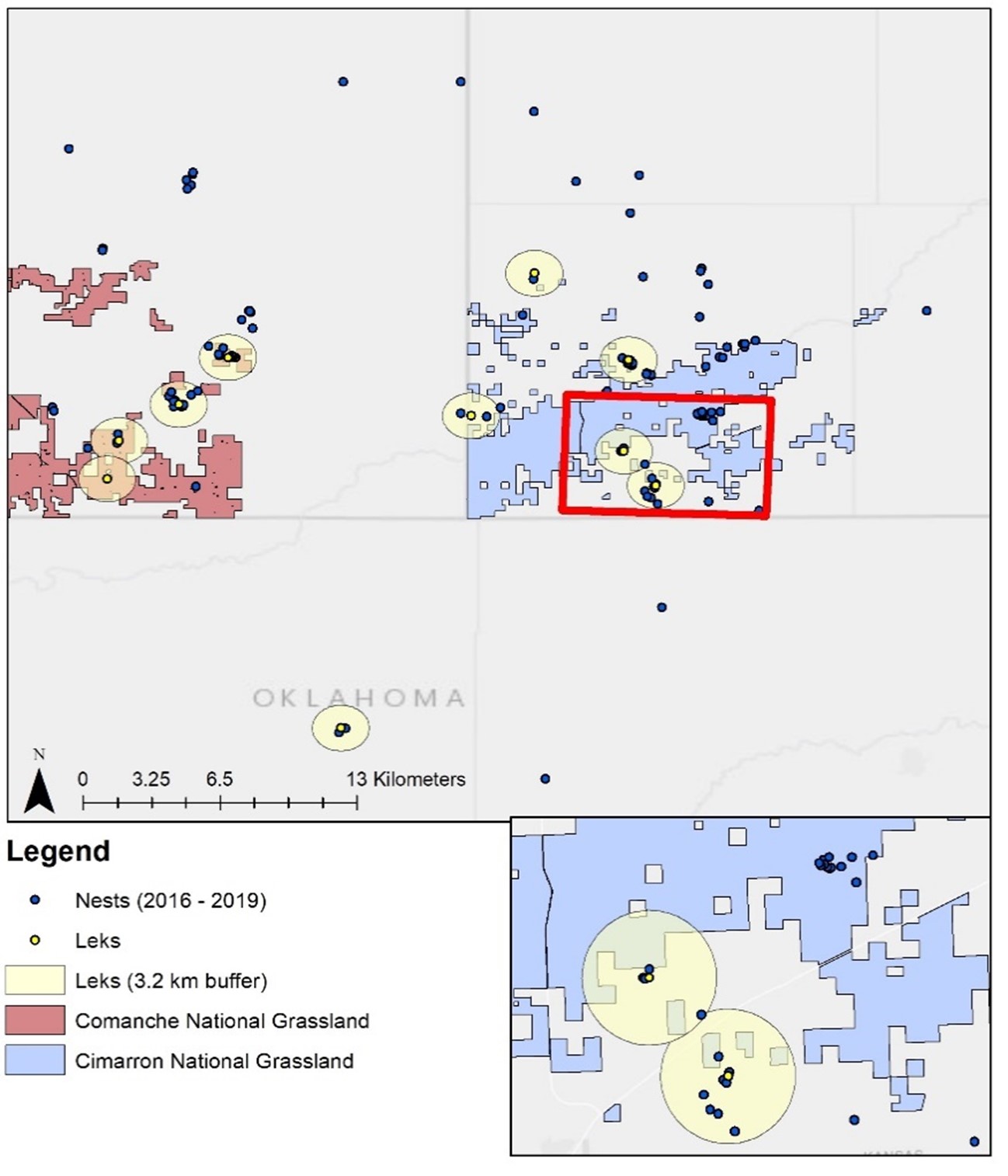

Supplement: Supplementary file 1 — Data S1 [file ECE3-14-e10871-s001.zip › ece310871-sup-0003-FigureS3.jpg]

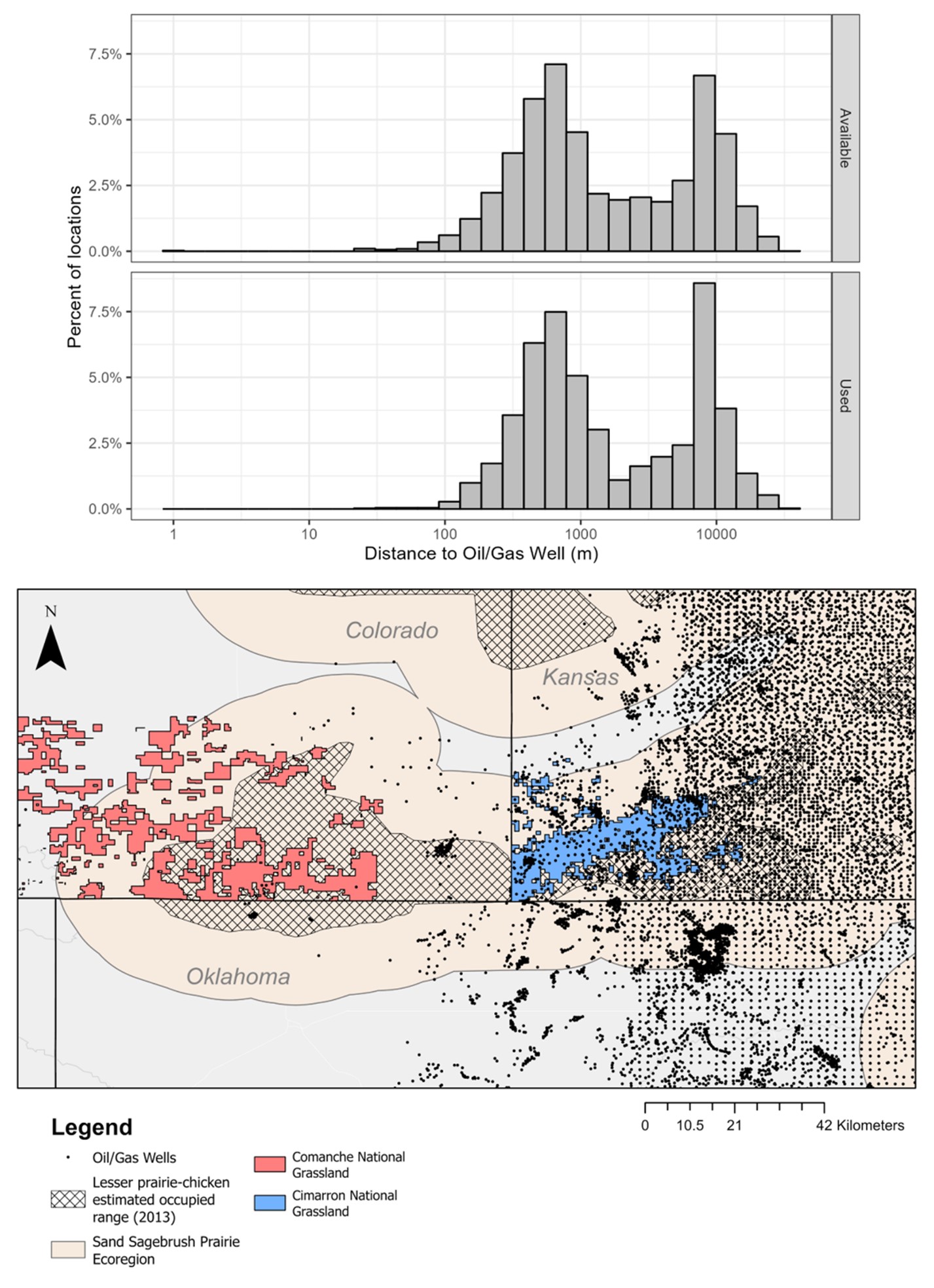

Supplement: Supplementary file 1 — Data S1 [file ECE3-14-e10871-s001.zip › ece310871-sup-0004-FigureS4.jpg]

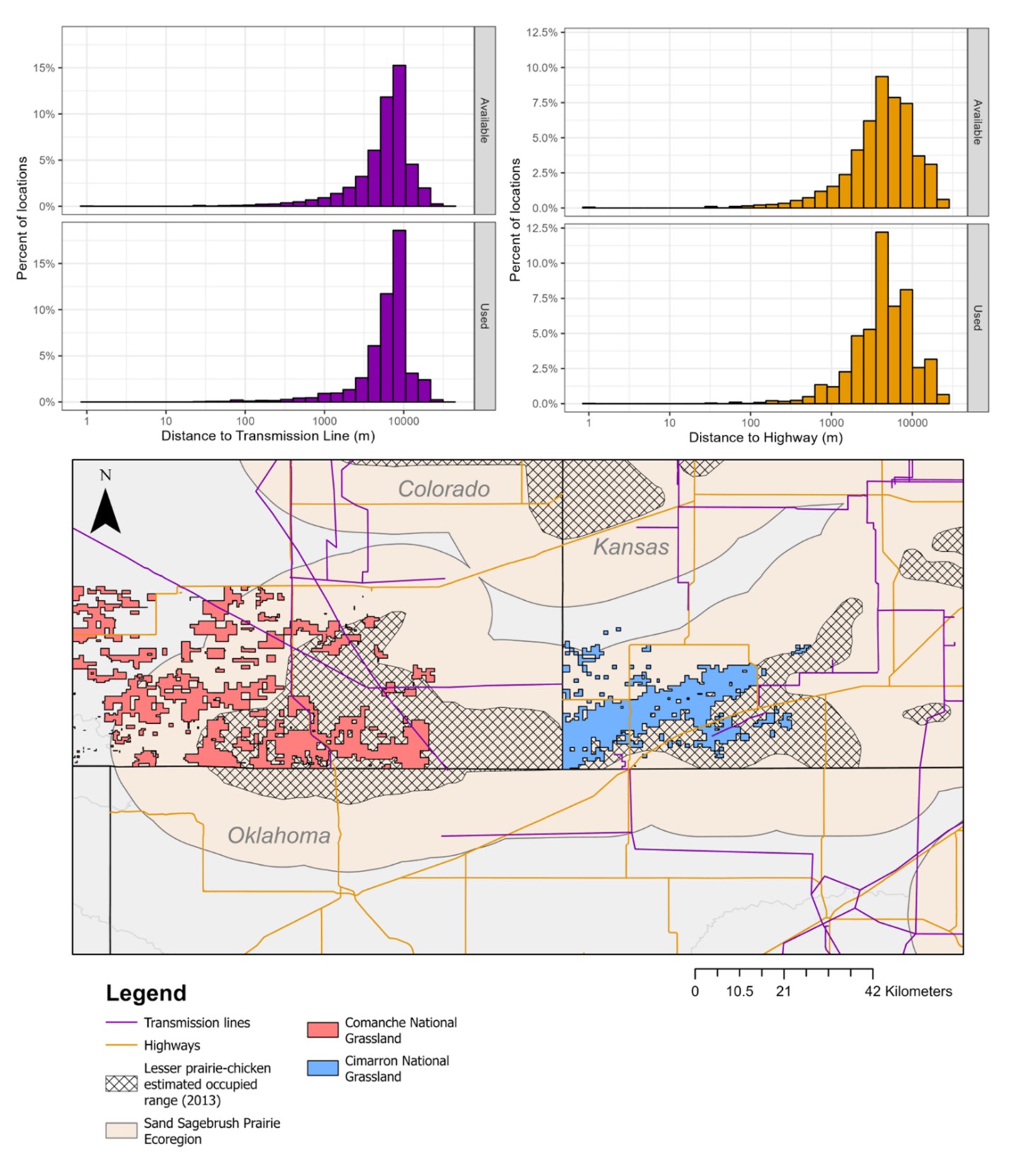

Supplement: Supplementary file 1 — Data S1 [file ECE3-14-e10871-s001.zip › ece310871-sup-0005-FigureS5.jpg]
